# Characteristics and outcomes of older patients undergoing out‐ versus inpatient surgery in Europe. A secondary analysis of the Peri‐interventional Outcome Study in the Elderly (POSE)

**DOI:** 10.1111/aas.70021

**Published:** 2025-03-24

**Authors:** Linda Grüßer, Mark Coburn, Matthias Schmid, Rolf Rossaint, Sebastian Ziemann, Ana Kowark, the POSE Study group, Alina Schenk, Ralf‐Dieter Hilgers, Federico Bilotta, Leo C Bollheimer, Wolfgang Buhre, Ulf Guenther, Andreas Hoeft, Peter Lee, Idit Matot, Steffen Rex, Jacob Steinmetz, Jos Tournoy, Zekeriyya Alanoglu, Marc M Berger, Xavier Falières, Nicolai Goettel, Andrijan Kartalov, Konstantinos Katsanoulas, Jakub Kenig, Victoria Khoronenko, Lars H Lundstrøm, Tamar Macharadze, Miodrag Milenovic, Serge Molliex, Rosário Órfão, Marina Soro, Mihai Stefan, Zerrin Sungur, Tamas Szakmany, Victoria Baños, Mireia Rodriguez, Selene Martinez, Thomas Saller, Simon T Schäfer, Edouard Clermond, Charlotte Martin, Charlene Le Moal, Frederik Staikowsky, Bertand Delannoy, Olivier Desebbe, Carlo Missant, Matthias Desmet, Hans‐Joerg Gillmann, Thomas Stueber, Sille M Dalsø, Morten Vester‐Andersen, Andreas Ranft, Gerhard Schneider, Christel Huygens, Roselien Meeusen, Patricia Cruz, Carmen Fernández, Mareike Otto, Agathe Giltaire, Pascal Hofmann, Simone Gurlit, Alejandro Romero Fernández, Federica Castelli, Alexandre Ntouba, Julien Lanoiselée, Regina Schulz, Mathias Opperer, Julia Van Waesberghe, Sebastian Ziemann, Einat Refaeli‐Awin, Zuleyha Kazak Bengisun, Immanuel Buchman, Dana Yahav‐Shafir, Antonia Dimakopoulou, Morgan Le Guen, Aurelio Rodríguez‐Pérez, Maud Beran, Aurelien Bonnal, Matthias Garot, Olivier Maupain, Denis Michel, Sofia Fernandes, Maria Sanabra, Eitan Mangoubi, Emmanuel Boselli, Timothy Switzer, I García‐Sánchez Jose, Matthieu Boisson, Konstantinos Stamoulis, María Merino García, Hinnerk Wulf, David Gouraud, Christophe Lebrun, Sigismond Lasocki, Luzius A Steiner, Lars Bergmann, Bertram Baenziger, Georgios Karpetas, Basak C Meco, Ayşe Hızal, Rosa Méndez Hernández, Valerie Smit‐Fun, Pedro Charco, Frank Nickel, Laura Grau Torradeflot, Marc Berger, Helmut Farcher, Mathias Opperer, Ine Adriaensens, Vera Saldien, Johan Berghmans, Sofie Van Hove, Maud Beran, Gert‐Jan Eerdekens, Dieter Mesotten, Maxim Timmers, Elly Vandermeulen, Ann De Bruyne, Stefan De Hert, Hendrik De Ruyter, Vincent Van Belleghem, Isabelle Boscart, Wouter De Corte, Matthias Desmet, Carlo Missant, Stefaan Carlier, Charlotte Castelain, Caroline Demeyer, Carl Vandenbossche, Carlo Missant, Hans Detienne, Sarah Devroe, Geertrui Dewinter, Danny Hoogma, Christel Huygens, Roselien Meeusen, Steffen Rex, Marc Van de Velde, Christophe Lebrun, Stéphanie Poels, Filiep Soetens, Christian Fenger‐Eriksen, Christina Draegert, Sofia Gaspar Santos, Christine Soelling, Jacob Steinmetz, Gertrud Andersen, Sille M Dalsø, Pernille Haderslev, Vibe M Rasmussen, Morten Vester‐Andersen, Tine G Sommer, Johan Kirkegaard, Lars H Lundstrøm, Christian M Olesen, Sansu Paramanathan, Lisbet Tokkesdal Jensen, Halfdan H Knudsen, Jens C Schmidt, Nick P Stehen, Hervé Dupont, Clément Herbinet, Emmanuel Lorne, Yazine Mahjoub, Alexandre Ntouba, Marine Fritsch, Manuela Garcia, Sigismond Lasocki, Jonathan Petit Phan, Thomas Lieutaud, Laura Bonneric, Emmanuel Boselli, Maxime Gaillet, Marc Danguy des Déserts, Etienne Montelescaut, Antoine Lamblin, Violaine Muller, Celine Lagrange, Charlene Le Moal, Alain Robert, Frederik Staikowsky, Benoit Lebas, Gilles Lebuffe, Matthias Garot, Johanne Beuvelot, David Dejour, Emmanuel Deligne, Olivier Desebbe, Bertand Delannoy, Benoit Gignoux, Olivier Guillaud, Joseph Nloga, Florence Prunier‐Bossion, Franck Sibellas, Paul Abraham, Cyril Bidon, Thomas Rimmele, Marie‐Hélène Bruge‐Ansel, Arnaud Friggeri, Anne‐Claire Lukaszewicz, Mikhail Dziadzko, Marc Leone, Zoe Meresse, Bruno Pastene, Isabelle Odin, Aurelien Bonnal, Nicolas Bouic, Pierre Trinh Duc, Thomas Pillant, Fabien Riboulet, Samuel Degoul, Nicolas Saumier, Marion Wasilewski, Karim Asehnoune, Antoine Roquilly, Pauline Glasman, Louis Puybasset, Fanny Garnier, Franck Verdonk, Charles M Samama, Line Towa, Alice Blet, Stéphanie Barrau, Matthieu Boisson, Bertrand Debaene, Denis Frasca, Nadia Imzi, Bernard Delvaux, Davy Huynh, Olivier Maupain, Luc Mercadal, Nabil Zanoun, Armelle de Baene, Catherine Boulay‐Maninovsky, Olivier Fernandes, Agathe Giltaire, Philippe Gomis, Jean‐Marc Malinovsky, François‐Xavier Romain, Astrid Calmelet, Ségolène Dupont, David Gouraud, Sophie Millet, Frédéric Simonneau, Francoise Charret, Charlène Couturier, Julien Lanoiselée, Estelle Lornage, Jeremy Mallard, Ryan Milati, Sylvie Passot, Sylvain Vallier, Mihaela L Agavriloaia, Quentin Badoux, Mehdi Lewandowski, Yanis Mermet, Denis Michel, Olga Kiskira, Sherifa Adjavon, Virginie Dumans, Morgan le Guen, Julien Josserand, Sabrina Ma, Jeremy Castanera, Benjamin Massiera, Philippe Petua, Fanny Bounes‐Vardon, Gaëlle Bosc, Laëtitia Bosch, Edouard Clermond, Fabrice Ferre, François Labaste, Charlotte Martin, Rémi Menut, Vincent Minville, Mohamed Srairi, Maria Tarasi, Florent Varin, Société Française d'Anaesthésie et de Réanimation (SFAR) Research Network, Paris, Mark Coburn, Ana Kowark, Linda Grüßer, Rolf Rossaint, Julia Van Waesberghe, Sebastian Ziemann, Lars Bergmann, Hartmuth Nowak, Günther Oprea, Katharina Rump, Matthias Unterberg, Heike Vogelsang, Mitja Klutzny, Claudia Neumann, Martin Soehle, Maria Wittmann, Martin Scharffenberg, Jakob Wittenstein, Jonas Hinterberg, Peter Kienbaum, Giovanna Lurati‐Buse, Frank Nickel, Maximilian Schäfer, Simone Lindau, Patrick Meybohm, Hans‐Joerg Gillmann, Florian Piekarski, Theresa A Kaufhold, Wolfgang Koppert, Andreas Leffler, Hans‐Peter Reiffen, Diana Rudolph, Henning Starke, Thomas Stueber, Petra Bischoff, Heinz Haberecht, Heiko Plehn, Michael Bauer, Andreas Kortgen, Christoph Sponholz, Uwe Krüger, Sabine Müller‐Esch, Mareike Otto, Christian Rempf, Christian Schmidt, Dunja Schumacher, Juliane Blazek, Christin Büttner, Andrea Leibeling, Dirk Rüsch, Hinnerk Wulf, Karsten Burow, Eugen A El‐Hilali, Christian Greke, Paul Großmann, Mario Kluth, Regina Schulz, Sofiane Dridi, Ivana Popovska, Andrés Brenes, Andreas Ranft, Pia Feddersen, Dominik Gerstmeyer, Philippe Fthenakis, Gerhard Schneider, Dirk Miketta, Vera von Dossow, Philipp Groene, Dominik Höchter, Klaus Hofmann‐Kiefer, Tobias Kammerer, Malte Kamrath, Thomas Saller, Simon T Schaefer, Roland Tomasi, Tobias Wiedemann, Catharina Zeuzem‐Lampert, Bernhard Zwissler, Stephan Braune, Mona Brune, Simone Gurlit, André Hemping‐Bovenkerk, Michael Möllmann, Mario Santamaria, Leonie M Schirwitz, Melanie Meersch, Alexander Zarbock, Ulf Guenther, Stefanie Decker, Berthold Drexler, Silvia Hipp, Pascal Hofmann, Markus Müller, Judith Roth, Miriam Seiß, Christian Adam, Ingo Schwartges, Peter Kranke, Konstantinos Katsanoulas, Pelagia Chloropoulou, Antonia Andreeva, Antonia Dimakopoulou, Amalia Douma, Iphigeneia Gregoriadou, Evelina Koutsouli, Konstantina Mendrinou, Eirini Mavrommati, Anastasios Stathopoulos, Chrysanthi Batistaki, Paraskevi Matsota, Konstantina Kalopita, Vasiliki Skandalou, Marina Balanika, Georgios Papathanakos, Petros Tzimas, Evgenia Ketikidou, Anastasia Vachlioti, Bioulent Kiamiloglou, Evangelia Nikouli, Eleni Arnaoutoglou, Konstantina Kolonia, Eleni Laou, Konstantinos Stamoulis, Epaminondas Vlachakis, Georgios Karpetas, Ioanna Lianou, Maria Spyraki, Irini Tatani, Eleni Panagiotou, Evangelia Samara, Anna Kolesnikova, Freideriki Sifaki, Eirini Zarzava, Athanasios Bampzelis, Eleni Georgopoulou, Eleni Christidou, Georgia Tsaousi, Maria Nastou, Orestis Ioannidis, Eugene Dolzenko, Georgia Geleve, Eleni Logotheti, Fotios Yfantidis, Peter Lee, Senbagam Rajamanickam, Shanmuga Ramaswamy, Timothy Switzer, Gurmukh Das Punshi, Karthikeyan Srinivasan, Michael Gilmartin, Osmond Morris, Immanuel Buchman, Yaacov Gozal, Amar Merissat, Reut Peled, Dafna Willner, Hila A Chariski, Leonid A Eidelman, Michal Y Livne, Eitan Mangoubi, Haim Berkenstadt, Dina Orlcin, Dana Yahav‐Shafir, Rita Aharonov, Anat Cattan, Lior Felman, Idit Matot, Einat Refaeli‐Awin, Yohai Steinberg, Wisam Zabeeda, Andrijan Kartalov, Biljana Kuzmanovska, Filip Naumovski, Marija Toleska, Atanas Sivevski, Xavier Falières, Anouk Andriessen, Minke Kortekaas, Wolfgang Buhre, Roos Van Gorp, Dianne Korte‐de Boer, Valerie Smit‐Fun, Maurice Theunissen, Mirjam Droger, Toine van den Enden, Seppe Koopman, Marije Marsman, Eva van Schaik, Jakub Kenig, Marta Azenha, Camile Lanzaro, Rosário Órfão, Andreia Borrego, Pedro Branquinho, Sofia Fernandes, Miguel Laires, Denise de Noronha, Inês Ferraz, Ana Pires, Joana Silva, Dan Corneci, Oana Oprea, Stefan‐Vladimir Zahiu, Dana R Tomescu, Ioana M Grintescu, Daniela Filipescu, Mihai Stefan, Elena Stefanescu, Andrey Vazenin, Danil Baskakov, Victoria Khoronenko, Dmitry Tipisev, Ksenia Kozlova, Olivera Marinkovic, Ana Sekulic, Miodrag Milenovic, Marija Rajkovic, Marija Djukanovic, Jovanka Nikolic, Svetlana Sreckovic, Marina Stojanovic, Nebojsa Ladjevic, Jelena Jovicic, Dragana Unic‐Stojanovic, Biljana Stosic, Aleksandra Bulasevic, Marina Soro, Alma M Espinosa‐Moreno, I García‐Sánchez Jose, Beatriz Martín‐Vaquerizo, Clara Morandeira‐Rivas, Diana Zamudio, Victoria Baños, Mireia Rodriguez, Selene Martinez, Nerea Guadalupe, Gracia Herranz, Javier Baute, Vanesa Madrona, Roser de Jose, Jordi Miralles, Alfred Merten, Rolando Muñoz, Anabel Delgado, Victoria Moral, Aleix Carmona Blesa, Sara Espejo, Laura Grau Torredeflot, Alejandro Romero Fernández, Maria Sanabra, Pere Serra Pujol, Maria J Alvira Uribe, Astrid Alvarez Perez, Espedito Brunetto, Federica Castelli, Jorge Gonzalez Aguirre, Adriana Herivas Villar, Guido Munoz Rojas, Aleix Carmona Blesa, Natalia Montero, Víctor Baladrón González, Ángel Becerra‐Bolaños, Aurelio Rodríguez‐Pérez, Luis Santana‐Ortega, Vanessa Suárez‐Romero, María L Torres‐Machí, Javier Ferrero de Paz, Jose M Marcos‐Vidal, Ana Martín Garcia, María Merino García, Consuelo Rego Diaz, Ana Crespo Santiago, Lourdes Ferreira Laso, Felix Lobato Solores, Alba Burgos, Alberto Calvo, Patricia Cruz, Carmen Fernández, Ignacio Fernández, Ignacio Garutti, Fernando Higuero, David Martinez, Patricia Piñeiro, Sonia Expósito Carazo, Rosa Méndez Hernández, Mar Orts Rodríguez, Fernando Ramasco Rueda, Ane Abad‐Motos, Javier Ripollés‐Melchor, Carmen Pastor López, Pedro Charco, Sara Perez‐Palao, Laura Sancho‐Iñigo, Nasara Segura, Marina Soro, Esther Utrera, Ania Albinarrate, Ana M Fondarella, Lucia Gallego‐Ligorit, Luisa Lacosta Torrijos, Oliver Bandschapp, Andrea A Blum, Nicolai Goettel, Esther Seeberger, Luzius A Steiner, Alessandra E Thomann, Seraina Frei, Susan Hoehn, Bertram Baenziger, Giuliana Capaldo, Daniel Christ, Ramon Doerig, Daniel Hodel, Andreas Weiss, Lukas Witt, Philippe Schumacher, Dirk A Siebing, Zerrin Sungur, Zekeriyya Alanoglu, Seyma Orcan Akbuz, Zuleyha Kazak Bengisun, Baturay K Kazbek, Ulku C Koksoy, Engin Z Terzi, Hakan Yilmaz, Neslihan Alkis, Sanem Cakar Turhan, Basak C Meco, Konul Hajiyeva, Cigdem Yildirim Guclu, Jülide Ergil, Emine Unal Ceran, Menekse Ozcelik, Atik Bülent, Kilinc Gökhan, Kemal T Saracoglu, Bunyamin Kir, Kemalettin Koltka, Nükhet Sivrikoz, Pelin Corman Dincer, Nur Canbolat, Turkan Kudsioglu, Gaye Aydin, Ceren Aygün Mucuoglu, Duriye G Inal, Semih Kucukguclu, I Egilmez Ayse, Betul Kozanhan, Munise Yildiz, Hüseyin U Pinar, Başar Erdivanlı, Ayşe Hizal, Emre Karagöz, Hızır Kazdal, Abdullah Özdemir, Ayca Tas Tuna, Gamze Gulgun, Dolya Oleg

**Affiliations:** ^1^ Department of Anaesthesiology University Hospital RWTH Aachen Aachen North Rhine‐Westphalia Germany; ^2^ Department of Anaesthesiology and Intensive Care Medicine University Hospital Bonn Bonn North Rhine‐Westphalia Germany; ^3^ Department of Medical Biometry, Informatics and Epidemiology, Faculty of Medicine University of Bonn Bonn North Rhine‐Westphalia Germany

**Keywords:** ambulatory surgery, cognitive and functional outcomes, older patients, postoperative mortality

## Abstract

**Background:**

The number of older patients undergoing surgical procedures with anaesthesia care is projected to rise. In order to cope with the increased demand, the expansion of outpatient surgery may play a decisive role. We aim to investigate the characteristics and outcomes of the older outpatient population.

**Patients and Methods:**

The Peri‐interventional Outcome Study in the Elderly in Europe (POSE) was a prospective multicenter study investigating characteristics and outcomes in 9497 patients aged 80 years and older undergoing a procedure with anaesthesia care. This secondary analysis of the POSE data investigated characteristics, functional and cognitive outcomes, and mortality in the outpatient in comparison to the inpatient population. Functional status was assessed as independent, partially dependent, and totally dependent at baseline and 30 days postinterventional. Cognitive status was defined by the number of recalled words (0–3) in the Mini‐Cog test and brief cognitive screening at baseline and follow‐up.

**Results:**

Out of the 9497 older patients, 7562 were planned inpatients and 1935 planned outpatients. Older outpatients presented with fewer comorbidities and fewer medications than older inpatients and underwent minor procedures more often Their baseline functional status was more often independent, and they had a higher estimated probability of staying independent. Outpatients recalled three words at baseline and follow‐up more often than inpatients. The estimated 30‐day survival probabilities with 95% confidence intervals were 0.997 [0.994; 0.999] in the group with planned outpatient surgery and 0.948 [0.942; 0.953] with planned inpatient surgery.

**Conclusion:**

Our results indicate that functional and cognitive status at baseline and follow‐up were higher in planned outpatients than in planned inpatients. However, only short screening tools for the assessment of functional and cognitive status were used. Overall, outpatient interventions were associated with low mortality. Further research is recommended to develop scores that facilitate the identification of patients suitable for outpatient surgery.

**Editorial Comment:**

This secondary analysis of a prospectively collected cohort of elderly surgical cases in Europe describes case factors related to inpatient compared to outpatient surgical interventions. The findings show that inpatient or outpatient surgery selection is associated with different degrees of risk for important perioperative outcomes in this cohort.

## 1 INTRODUCTION

Worldwide, people aged 65 years and older are the fastest‐growing age group.^1^ In Europe, the number of older people aged 85 years and older is expected to increase by up to 130% from 2018 to 2050.^2^ These demographic changes suggest a sharp rise in the number of older patients undergoing surgical procedures with anaesthesia care. In order to cope with the increased demand in already overstrained health care systems, expansion of outpatient surgery may play a decisive role. Besides the economic benefits, it has been argued that older patients recover more rapidly from surgery within their familiar home environment with less disruption of their normal schedule.^3^ Furthermore, common risks of hospitalization, such as nosocomial infections, are reduced.^4^ However, little is known about the older outpatient population in Europe. It is unclear which older patients are currently being offered outpatient surgical care, and only a few studies have investigated outcomes after outpatient surgery.^5^ One international prospective multicentre cohort study in 372 older patients undergoing minor surgery revealed a lower incidence of postoperative cognitive dysfunction in older outpatients compared with inpatients.^6^ Of note, the prevalence rate of pre‐operative cognitive impairment in the older surgical outpatient population in the United States was already presented as slightly lower for the general and elective surgery population.^7^, ^8^, ^9^ Overall, studies investigating the advantages of enhanced recovery pathways on postoperative cognitive decline and functional status in the older patient population have shown shortened lengths of stay and beneficial outcomes.^10^, ^11^ However, these are not focused on outpatients.

The Peri‐interventional Outcome Study in the Elderly in Europe (POSE) was a prospective multi‐centre study investigating characteristics and outcomes in 9497 patients aged 80 years and older undergoing any kind of out‐ and inpatient surgical or nonsurgical procedure with anaesthesia care.^12^ The present secondary analysis of the POSE data is the first study that analyses the older European outpatient population. We aim to describe differences in the characteristics and outcomes in the outpatient in comparison to the inpatient population.

## 2 METHODS

### 2.1 Study design

We performed a secondary analysis of the prospective observational European multicentre POSE study.^12^ The original study was registered with ClinicalTrials.gov (NCT03152734), and the study protocol is available at www.pose-trial.org/study-documents. The study design has been described in detail previously.^12^ This secondary analysis was pre‐planned before the database closing, and the study proposal has been approved by the POSE Steering Committee.^13^ This work is reported in concordance with the STROBE statement. Following approval or a waiver from each respective centre's research ethics board (REB), written informed consent was obtained as required according to national laws.^12^ A separate REB approval for this secondary analysis was not required.

### 2.2 Setting, participants, and data collection

All analyses were based on the POSE study data. POSE analysed 9497 patients from 177 participating centres in 20 countries.^12^ Patients aged at least 80 years and undergoing any kind of surgical or nonsurgical intervention with anaesthesia care were included. For this secondary analysis, outpatients were defined as any patients having pre‐planned outpatient surgery with discharge on the day of intervention. Recruitment per centre took place during a 30‐day self‐selected period between October 2017 and December 2018. Data on the pre‐operative status, including medical conditions, cognitive and functional status, and intraoperative surgical and anaesthesia characteristics, were collected. Each patient was followed up for 30 days after the procedure. As previously described, all data was collected prospectively on paper‐based case report forms and entered into an electronic database (OpenClinica, Boston, Massachusetts, USA).^12^

### 2.3 Outcome measures and covariates

Based on the pragmatic case report form of the POSE study, the following outcome variables were analysed at baseline and follow‐up: Functional status (three categories: independent, partially dependent, and totally dependent^14^), cognitive status (defined at baseline by the number of recalled words (0–3) in the Mini‐Cog test,^15^ and—in order to enable a telephone‐follow up comparison—defined at follow‐up by the number of recalled words (0–3) in the brief cognitive screening by one item of the BSCI^16^) and time to death (ranging between 0 and 30 days, 388 observed deaths). Baseline data was collected in person at the hospital. Follow‐up was performed in person, in case the patient was still at the hospital, or via telephone, if the patient was already discharged.

The following nine covariates were identified as possible confounders: (1) age at baseline (years, modelled as continuous covariate with linear effect), (2) sex (male and female), (3) ASA classification (five categories, modelled as continuous covariate with linear effect), (4) kind of referring facility (home, other hospital, rehabilitation, nursing home, and other), (5) multimorbidity defined as the presence of at least two of the assessed comorbidities (yes and no), (6) timed up and go (TUG) test^17^ (limited mobility defined as TUG test performed in 12 s,^18^ normal), (7) history of falls (none, once, and more than once), (8) application of premedication (yes and no), (9) anaesthesia technique (general, regional, sedation, and combination). In addition, we considered severity of surgery (minor, intermediate, and major), urgency of surgery (elective, urgent, and emergency), and surgical category (abdominal, cardiovascular and thoracic, ears, nose and throat (ENT) and ophthalmic, gynaecologic and urologic, interventional, neurosurgery, orthopaedic, trauma and plastic, and other). However, we excluded these variables from analysis, as they were very strongly correlated with outpatient surgery or even determined the outpatient/inpatient status.

### 2.4 Bias

POSE aimed at minimizing selection bias and providing generalizable results by the consecutive recruitment of all eligible patients in a selected 30‐day recruitment period.^12^ Legally incompetent and emergency patients were also included. Measures to decrease the risk of attrition and detection bias were implemented.^12^

### 2.5 Statistical analysis

The statistical analysis was carried out on the basis of the cleaned and closed POSE database.^12^ Statistical analysis was performed using R, version 3.5.1 (R Foundation for Statistical Computing, Vienna, Austria). Continuous variables were summarized using means with SD and medians with the 25% and 75% quantiles. Categorical variables were summarized using counts with percentages.

Changes in functional status (baseline vs. follow‐up) were analysed using cross tabulations with McNemar's test. An ordinal logistic regression model with outcome variable ‘functional status at follow‐up’ and covariates ‘functional status at baseline’ and ‘outpatient vs. inpatient surgery’ (including an interaction term) was fitted to the analysis data. To adjust the analysis for the effects of possible confounder variables, we additionally fitted a multivariable ordinal logistic regression model with outcome variable ‘functional status at follow‐up’ and covariates ‘functional status at baseline’, ‘outpatient vs. inpatient surgery’ (including an interaction term between the two aforementioned covariates), and the nine covariates listed above to the analysis data. Likelihood ratio tests were used to compute *p*‐values for the effect of ‘outpatient vs. inpatient surgery’. *p* < .05 was considered statistically significant in all analyses. Missing values in the nine confounder variables were imputed using multiple imputation with 12 runs, as described elsewhere, and the model was fitted 12 times.^12^ Estimated probabilities of functional status at follow‐up were computed from these models by averaging predictions in the subgroups defined by ‘functional status at baseline’ and ‘outpatient vs. inpatient surgery’.

Changes in cognitive status (baseline vs. follow‐up) were analysed using cross tabulations with McNemar's test. Furthermore, a quasi‐Poisson regression model with outcome variable ‘number of recalled words at follow‐up’ and covariates ‘number of recalled words at baseline’ and ‘outpatient vs. inpatient surgery’ (including an interaction term) was fitted to the analysis data. To adjust the analysis for the effects of possible confounder variables, we additionally fitted a multivariable quasi‐Poisson regression model with outcome variable ‘number of recalled words at follow‐up’ and covariates ‘number of recalled words at baseline’, ‘outpatient vs. inpatient surgery’ (including an interaction term between the two aforementioned covariates), and the nine covariates listed above to the analysis data. Quasi‐likelihood ratio tests were used to compute p‐values for the effect of ‘outpatient vs. inpatient surgery’. Again, missing values were imputed using multiple imputation with 12 runs, and the model was fitted 12 times. Estimated means of the number of recalled words at follow‐up were computed from these models by averaging predictions in the subgroups defined by ‘number of recalled words at baseline’ and ‘outpatient vs. inpatient surgery’.

Time to death was analysed using Kaplan–Meier estimates (grouped by ‘outpatient vs. inpatient surgery’) with a log‐rank test. In addition, Cox regression models with covariate ‘outpatient vs. inpatient surgery’ and the nine possible confounders listed in Section 1 were fitted. The Cox model was also fitted 12 times using the imputed data sets. A 95% percent confidence interval for the pooled hazard ratio of ‘outpatient vs. inpatient surgery’ was computed using Rubin's rules.

For descriptive analysis, all missing values in the analysed variables were tabulated. Patients with a missing value in the outcome variables at baseline or follow‐up were excluded from the respective statistical analyses. Functional and cognitive status of patients who died during follow‐up were set to ‘totally dependent’ and ‘zero recalled words’ respectively.

## 3 RESULTS

### 3.1 Baseline and perioperative characteristics

Descriptive summaries of the variables at baseline and perioperative period are presented in Tables 1 and 2, respectively. There were no missing values in the binary variable with categories pre‐planned ‘outpatient vs. inpatient surgery’. Out of the 9497 patients, 7562 were planned as inpatients and 1935 as outpatients. There were no major differences between inpatients and outpatients with regard to age, sex, height, and weight. Overall, outpatients had less comorbidities and took fewer medications (Supplemental Table 1. Comorbidities and medication). This is also reflected within their ASA score. Outpatients were more often classified as ASA 1 and 2 compared with inpatients (56.7% vs. 34.0%). Fewer outpatients had a limited TUG test (55.5% vs. 71.2%) and reported ≥1 fall within the last 6 months (18% vs. 34.8%). In comparison to inpatients, outpatients' functional status was more often independent (74.0% vs. 58.4%), and they scored 4–5 points out of 5 possible points in the Mini‐Cog test more frequently (42.9% vs. 37.4%). They recalled three words at baseline more often (42.8% vs. 35.8%). The mean number of correctly recalled words was 2.0 (±1.1) in comparison to 1.8 (±1.1) in inpatients. Outpatients' referring facility was mostly from home (91.2% vs. 85.4%) and less often from a nursing home (3.8% vs. 7.9%) or other hospital (0.4% vs. 2.3%).

**TABLE 1:** Baseline characteristics of outpatients and inpatients.

	Outpatient (*n* = 1935)	Inpatients (*n* = 7562)	Overall (*n* = 9497)
Age (year)	83.0 (81.0–86.0)	84.0 (81.0–87.0)	83.0 (81.0–86.0)
Sex			
Male	911 (47.1%)	3574 (47.3%)	4485 (47.2%)
Female	1024 (52.9%)	3988 (52.7%)	5012 (52.8%)
Height (cm)	164 (157–170)	165 (159–171.0)	165 (158.0–170.0)
Weight (kg)	70.0 (61.0–79.0)	70.0 (60.0–80.0)	70.00 (60.0–80.0)
ASA score			
1	74 (3.8%)	96 (1.3%)	170 (1.8%)
2	1023 (52.9%)	2476 (32.7%)	3499 (36.8%)
3	808 (41.8%)	4298 (56.8%)	5106 (53.8%)
4	29 (1.5%)	663 (8.8%)	692 (7.3%)
5	0 (0%)	23 (0.3%)	23 (0.2%)
Referring facility			
Home	1765 (91.2%)	6455 (85.4%)	8220 (86.6%)
Other hospital	8 (0.4%)	176 (2.3%)	184 (1.9%)
Rehabilitation	3 (0.2%)	57 (0.8%)	60 (0.6%)
Nursing home	74 (3.8%)	596 (7.9%)	670 (7.1%)
Other	85 (4.4%)	275 (3.6%)	360 (3.8%)
Multimorbidity^a^	1319 (68.2%)	6015 (79.5%)	7334 (77.2%)
Haemoglobin (g dL^−1^)	13.1 (11.8–14.2)	12.4 (11.0–13.6)	12.5 (11.1–13.7)
History of falls during the last 6 months			
None	1567 (81.0%)	4859 (64.3%)	6426 (67.7%)
1 time	244 (12.6%)	1561 (20.6%)	1805 (19.0)
>1 time	105 (5.4%)	1076 (14.2%)	1181 (12.4%)
Limited timed up and go test^b^	1074 (55.5%)	5387 (71.2%)	6461 (68.0%)
Unintentional weight loss≥4.5kg in the last year	238 (12.3%)	1476 (19.5%)	1714 (18.0%)
Functional status at baseline			
Independent	1431 (74.0%)	4414 (58.4%)	5845 (61.5%)
Partially dependent	430 (22.2%)	2473 (32.7%)	2903 (30.6%)
Totally dependent	73 (3.8%)	670 (8.9%)	743 (7.8%)
Mini‐Cog^c^: Total points			
0	180 (9.3%)	1212 (16.0%)	1392 (14.7%)
1–3	812 (42.0%)	3189 (42.2%)	4001 (42.1%)
4–5	830 (42.9%)	2831 (37.4%)	3661 (38.5%)
Mini‐Cog − mean number of correctly recalled words	2.01 ± 1.05	1.80 ± 1.12	1.84 ± 1.11
Mini‐Cog − mean number of clock draw points	1.11 ± 0.995	0.960 ± 0.999	0.989 ± 1.00

*Note*: Data are presented as *n* (%), mean ± SD or median (IQR). Missing data on height *n* = 146, 1.5% (outpatients *n* = 40, 2.1%; inpatients *n* = 106, 1.4%); weight *n* = 97, 1.0% (outpatients *n* = 27, 1.4%; inpatients *n* = 70 (0.9%)); ASA *n* = 7, 0.1% (outpatients *n* = 1, 0.1%; inpatients *n* = 6, 0.1%); referring facility *n* = 3, 0.0% (outpatients *n* = 0, 0.0%; inpatients *n* = 3, 0.0%); haemoglobin not measured/missing *n* = 1863, 19.6% (outpatients *n* = 1116, 57.7%, inpatients *n* = 747, 9.9%); history of falls *n* = 85, 0.9% (outpatients *n* = 19, 1.0%; inpatients *n* = 66, 0.9%); Timed‐up and go test *n* = 1126, 11.9% (outpatients *n* = 305, 15.8%; inpatients 821, 10.9%); unintentional weight loss *n* = 104, 1.1% (outpatients *n* = 22, 1.1%; inpatients *n* = 82, 1.1%); functional status at baseline *n* = 6, 0.1% (outpatients *n* = 1, 0.1%; inpatients *n* = 5, 0.1%); Mini‐Cog total points *n* = 443, 4.7% (outpatients *n* = 113, 5.8%; inpatients *n* = 330, 4.3%); Mini‐Cog number of recalled words *n* = 373, 3.9% (outpatients *n* = 70; 3.6%, inpatients *n* = 303, 4.0%); Mini‐Cog clock draw points *n* = 442, 4.7% (outpatients *n* = 113, 5.8%, inpatients *n* = 329, 4.4%).

^a^ Multimorbidity was defined as the presence of at least two of the assessed comorbidities.

^b^ Timed up and go test was defined as limited when performed in >12 s.

^c^ Mini‐Cog screening tool to detect cognitive impairment or dementia: 0 = profound cognitive dysfunction, ≤3 = cognitive impairment according to Robinson et al.,^31^ 5 = normal cognition.

**TABLE 2:** Perioperative characteristics of inpatients and outpatients.

	Outpatients (*n* = 1935)	Inpatients (*n* = 7562)	Overall (*n* = 9497)
Urgency			
Elective	1891 (97.7%)	5285 (69.9%)	7176 (75.6%)
Urgent	33 (1.7%)	1809 (23.9%)	1842 (19.4%)
Emergency	11 (0.6%)	468 (6.2%)	479 (5.0%)
Severity of surgery			
Minor	895 (46.3%)	1052 (13.9%)	1947 (20.5%)
Intermediate	991 (51.2%)	2621 (34.7%)	3612 (38.0%)
Major	49 (2.5%)	3889 (51.4%)	3938 (41.5%)
Type of intervention				
Abdominal	62 (3.2%)	1087 (14.4%)	1149 (12.1%)
Cardiovascular and thoracic	47 (2.4%)	849 (11.2%)	896 (9.4%)
ENT and ophthalmic	970 (50.1%)	624 (8.3%)	1594 (16.8%)
Gynaecologic and urological	218 (11.3%)	1219 (16.1%)	1437 (15.1%)
Interventional	327 (16.9%)	699 (9.2%)	1026 (10.8%)
Neurosurgery	3 (0.2%)	193 (2.6%)	196 (2.1%)
Orthopaedic, trauma and plastic	233 (12.0%)	2627 (34.7%)	2860 (30.1%)
Other surgery	75 (3.9%)	264 (3.5%)	339 (3.6%)
Laparoscopic surgery	28 (1.4%)	522 (6.9%)	550 (5.8%)
Cancer surgery	132 (6.8%)	1294 (17.1%)	1426 (15.0%)
Median anaesthesia duration (min)	33.0 (23.0–55.0)	105.0 (65.0–160)	90.00 (48.0–142)
Anaesthesia technique				
General	438 (22.6%)	4614 (61.0%)	5052 (53.2%)
Regional^a^	289 (14.9%)	1339 (17.7%)	1628 (17.1%)
Sedation	1021 (52.8%)	734 (9.7%)	1755 (18.5%)
Combination^b^	187 (9.7%)	875 (11.6%)	1062 (11.2%)
Premedication before the intervention^c^	463 (23.9%)	1088 (14.3%)	1551 (16.3%)
Application of safe surgery checklist	1397 (72.2%)	5682 (75.1%)	7079 (74.5%)
Transfusion of platelets	0 (0%)	64 (0.8%)	64 (0.7%)
Transfusion of plasma	0 (0%)	141 (1.9%)	141 (1.5%)
Transfusion of RBC	2 (0.1%)	573 (7.6%)	575 (6.1%)
ICU admission	11 (0.6%)	1646 (21.8%)	1657 (17.4%)
Admission to geriatric support unit	18 (0.9%)	661 (8.7%)	679 (7.1%)
Mean hospital length of stay (days)	0.173 ±1.60	7.31 ± 7.48	5.86 ±7.31

*Note*: Data are presented as *n* (%), mean ± SD or median (IQR). Missing data median anaesthesia duration *n* = 22, 0.2% (outpatients *n* = 2, 0.1%; inpatients *n* = 20, 0.3%), premedication before the intervention *n* = 10, 0.1% (outpatients *n* = 1, 0.1%; inpatients *n* = 9, 0.1%); application of safe surgery checklist *n* = 16, 0.2% (outpatients *n* = 5, 0.3%; inpatients *n* = 11, 0.1%); transfusion of platelets *n* = 1, 0.0% (outpatients *n* = 0, 0.0%; inpatients *n* = 1, 0.0%); transfusion of plasma *n* = 1, 0.0% (outpatients *n* = 0, 0.0%; inpatients *n* = 1, 0.0%); transfusion of RBC *n* = 1, 0.0% (outpatients *n* = 0; 0.0%, inpatients *n* = 1, 0.0%); ICU admission *n* = 1, 0.0% (outpatients *n* = 0; 0.0%, inpatients *n* = 1, 0.0%); Geriatric support unit *n* = 1, 0.0% (outpatients *n* = 0, 0.0%, inpatients *n* = 1, 0.0%).

Abbreviations: ENT, ears, nose and throat; ICU, intensive care unit; RBC, red blood cells.

^a^ Regional anaesthesia includes epidural, spinal, or other regional anaesthesia techniques.

^b^ Combined anaesthesia is defined as a combination of at least two of the three given categories: general anaesthesia, sedation, or regional anaesthesia.

^c^ Premedication comprises benzodiazepines and clonidine.

The type of intervention and the anaesthesia technique differed between the outpatient and inpatient populations. The most common types of interventions in outpatients were ENT (50.1% vs. 8.3%) and non‐surgical interventions (16.9% vs. 9.2%). Overall, in comparison to inpatients, outpatients more often had minor (46.3% vs. 13.9%) or intermediate procedures (51.2% vs. 34.7%), and the procedure was more often elective (97.7% vs. 69.9%). Outpatients underwent the respective interventions more often in sedation (52.8% vs. 9.7%). Accordingly, their median anaesthesia duration was shorter (33.0 min (23.0–55.0) vs. 105 min (65–160)).

Out of 1935 patients who were originally planned for an outpatient procedure, 101 patients (5.2%) eventually received inpatient care. The mean and median [min to max] length of stay for outpatients versus inpatients was 0.2 (±1.6) and 0 [0 to 31] versus 7.3 (±7.5) and 5 [0 to 31] days, respectively. There were 11 planned outpatients (0.6%) who were admitted to an ICU versus 1646 (21.8%) planned inpatients.

### 3.2 Outcomes

#### 3.2.1 Functional status

**FIGURE 1:**
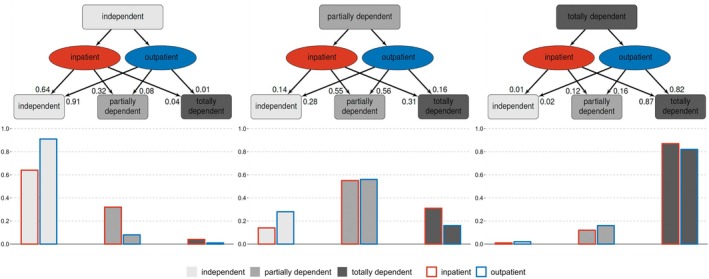
Functional status. Figure showing the estimated probabilities of the categories of functional status at follow‐up (obtained from ordinal logistic regression).

Out of the 9497 patients in the POSE dataset, 8965 patients were included for the functional status analysis (Supplemental Table 2). Cross tabulations of functional status (baseline vs. follow‐up, all patients + grouped by outpatients vs. inpatients) are presented in **S**upplemental Table 3. *p*‐values of McNemar's test were<0.001 throughout, indicating significant changes. The estimated probabilities of functional status categories at follow‐up obtained from ordinal logistic regression are presented in Figure 1. In comparison to inpatients, outpatients had a higher estimated probability to stay independent (0.91 vs. 0.64).

The multivariable ordinal logistic regression models yielded similar results (Supplemental Table 4). The estimated probability for independent outpatients to maintain an independent functional status was 0.91 versus 0.63 for inpatients. Additionally, when removing the covariate ‘outpatient vs. inpatient surgery’ from the model, *p*‐values remained <0.001 in all 12 imputations, indicating a strong effect of this variable on functional status at follow‐up.

#### 3.2.2 Cognitive outcome

**FIGURE 2:**
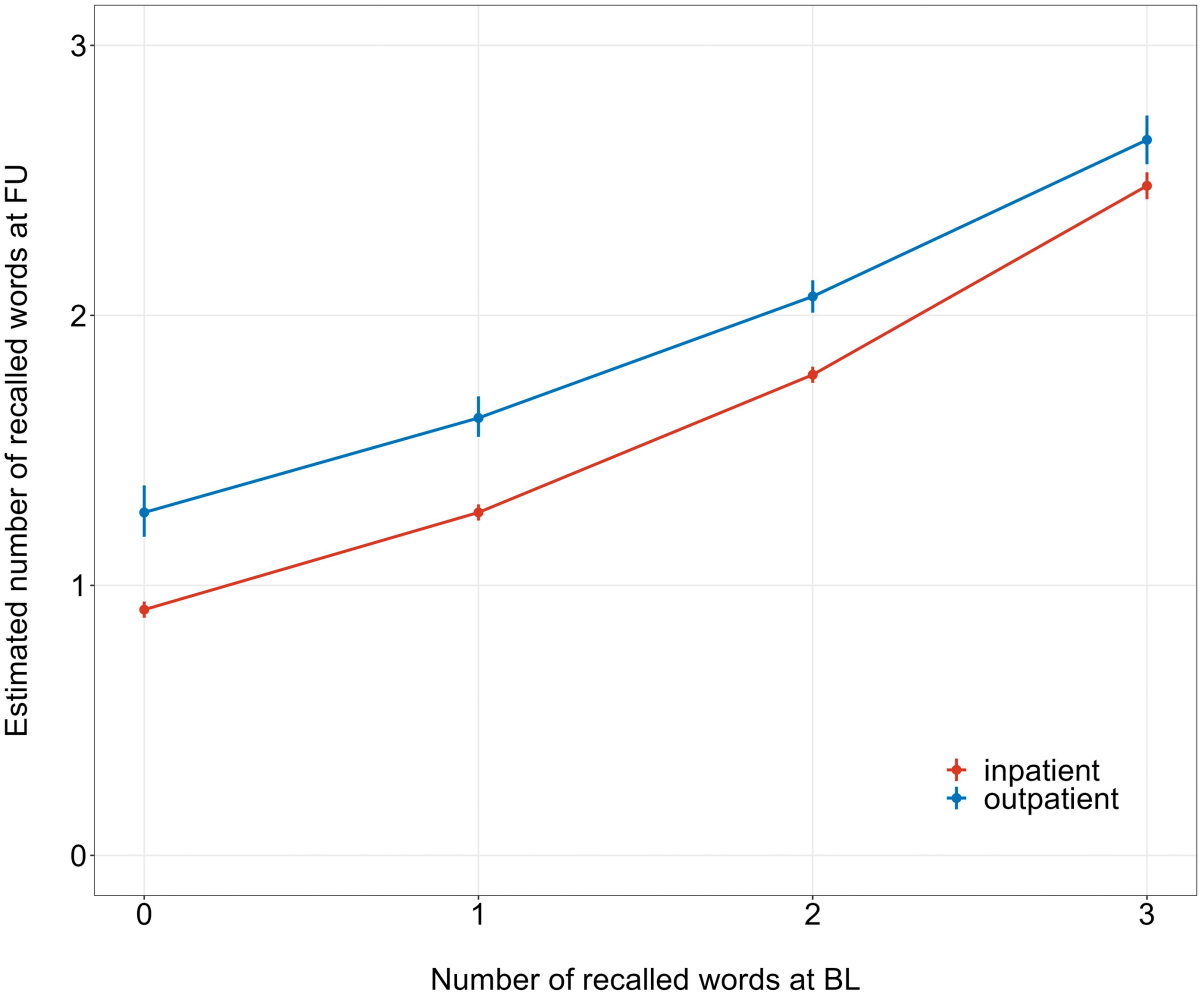
Number of recalled words. Figure showing the estimated means of the number of recalled words at follow‐up (quasi‐Poisson regression).

Out of the 9497 patients, 8015 were included in the cognitive outcome analysis (Supplemental Table 2). Cross tabulations of cognitive status (baseline vs. follow‐up, all patients + grouped by outpatient vs. inpatient surgery) are presented in Supplemental Table 5. *p*‐values of McNemar's test were<0.001 throughout, indicating significant changes. The estimated number of recalled words at follow‐up was 2.15 for outpatients versus 1.78 for inpatients. The estimated means of the number of recalled words at follow‐up (obtained from quasi‐Poisson regression) are presented in Figure 2.

The estimated means of the number of recalled words at follow‐up obtained from multivariable quasi‐Poisson regression are presented in Supplemental Table 6. Removing the covariate ‘outpatient vs. inpatient surgery’ from the model resulted in *p*‐values that were<0.001 in all 12 imputations, indicating a strong effect of this variable on functional status at follow‐up.

#### 3.2.3 Mortality

We observed 382 deaths in the inpatient group and 6 deaths in the outpatient group. The estimated 30‐day survival probabilities with 95% CI were 0.997 [0.994–0.999] in the group with planned outpatient surgery and 0.948 [0.942–0.953] in the group with planned inpatient surgery. Regarding time to death, the log‐rank test resulted in a *p*‐value <.001, indicating a strong effect of planned outpatient versus planned inpatient status on survival. In Cox regression, the planned outpatient versus inpatient status was strongly associated with survival, reducing the hazard of death by more than 80% after adjusting for possible confounding (pooled hazard ratio = 0.161 [0.071–0.366]).

Additional outcomes are presented in Supplemental Table 7.

## 4 DISCUSSION

To the best of our knowledge, this study is the first large‐scale analysis of older outpatients' characteristics and outcomes in Europe. Older outpatients presented with fewer comorbidities and fewer medications than older inpatients and underwent more often minor procedures. Our results indicate that functional and cognitive status at baseline were higher in outpatients than in inpatients, and the risk of postoperative deterioration was lower. Overall, outpatient interventions were associated with low mortality.

Weighing benefits against risks of interventions is difficult, particularly in the older patient population, but is likely to become a more frequent challenge in the near future. Besides survival, patient‐centred outcomes have come to the fore. Seriously ill older patients may not choose treatment for survival if that would go along with severe functional or cognitive impairment.^19^ Zhang et al. found that more than 20% of older inpatients who underwent a non‐orthopaedic surgical procedure experienced a functional decline.^20^ In comparison, the estimated probability for an independent older patient to maintain independent functional status was 0.91 in our outpatient population. The risk of becoming totally dependent was 0.01. In general, independent or partially dependent outpatients had higher probabilities to maintain or to improve their functional status in comparison to inpatients. For older patients who were already totally dependent at baseline, the differences were not as evident.

Similarly, we observed that outpatients had higher cognitive scores at baseline and at follow‐up. Thus, our results are in line with the scarce available evidence on cognitive outcomes after outpatient surgery.^5^, ^6^ It has repeatedly been shown that age is associated with adverse outcomes after surgery and that, in particular, older patients with preoperative cognitive impairment are vulnerable.^21^, ^22^ In our outpatient population, 50% of outpatients with 0 recalled words at baseline improved the number of recalled words at follow‐up. However, there may be considerable variations in the degree of cognitive impairment within a category, in particular within the ‘0 recalled words category’. It is crucial to underline that both functional and cognitive outcome measures in our analysis are based on very simple and short assessments that were part of the POSE study and not on recommended elaborate questionnaires such as the WHO Disability Assessment Schedule questionnaire (WHODAS).^23^, ^24^ It is unknown to what degree the classification of the functional status according to the ACS‐NSQIP used in our study correlates with the WHODAS or if recalling of words truly presents a rough surrogate for assessing changes in pre‐ and postoperative cognitive function.

Nevertheless, we detected differences between the two patient populations, confirming what has been suspected from daily clinical routine: Older patients who are being offered outpatient surgery are usually fitter, undergo less severe interventions, and have better outcomes. It is hence not surprising that the severity of surgery, urgency of surgery, and surgical category strongly correlated with or even determined the pre‐planned in‐ and outpatient status. Consequently, it is not possible to conclusively determine the extent to which the outpatient status or the type of intervention contributed to the positive outcome. What we can observe is that anaesthesiologists' clinical decisions appear to have identified a group of patients with similar characteristics, for whom outpatient surgery was an appropriate choice. Notably, 101 pre‐planned outpatients (5.2%) had a hospital stay longer than 1 day versus 44 pre‐planned inpatients (0.6%) who were discharged on the day of the procedure. Pre‐planned outpatients who had an unplanned hospital stay had a mean and median length of hospital stay of 3.32 ± 6.24 and 1 (1‐2) days, respectively. In comparison to pre‐planned outpatients who stayed outpatients, they underwent more often major surgeries and received more often a general anaesthesia than a sedation (Supplemental Table 8). Their baseline cognitive and functional status were lower, but their sample size was too small to derive meaningful predictors for outcomes or a successful outpatient status. Developing scores and guidelines that facilitate the identification of patients suitable for outpatient surgery is essential to enhance patient safety and efficiency.^25^, ^26^ Larger study populations and multicentre ranomised controlled trials are needed to build and validate such scores for older patients. Further research should also focus on the development of postoperative outpatient support systems, such as telemedical support. Embracing patient empowerment concepts, the decision of whether an older patient is suited for outpatient surgery should always be made together with the patient, considering their domestic environment and availability of family member support. The shared decision model has been proposed to incorporate an individualized approach to perioperative care.^27^, ^28^

Our study comes along with several limitations. First and foremost, this is a secondary analysis of the POSE study and not a study designed for investigating functional and cognitive outcomes among outpatients and inpatients.^12^ Questions relating to the cognitive and functional status were restricted to a minimum to enable voluntary participation of 177 study centres across Europe. Our multivariable analysis accounted for important variables associated with functional and cognitive outcomes, but we acknowledge the risk of unmeasured confounding, for example, we could not adjust for years of education or social determinants. Even though we adjusted for the kind of anaesthesia, the effects of different anaesthesia regimes on cognitive decline in older patients require further investigation.^29^ Also, we did not adjust for random centre effects or differences among the national health care systems. In general, limitations of the POSE data collection apply to our analysis as well.^12^ It is important to consider that participating POSE centres were mainly tertiary or academic secondary hospitals.^12^ It remains unclear whether older outpatients in Europe are generally more likely to undergo outpatient surgery in such a setting or if a considerable proportion also undergo interventions with anaesthesia care in primary care facilities. Older patients receiving outpatient care in primary care facilities may have even fewer comorbidities and favourable outcomes. Overall loss‐to‐follow‐up rates in the main POSE study can be considered low.^12^, ^30^ Nevertheless, it could be argued that patients who died during the follow‐up period should not have been classified in the lowest category of the respective outcomes. However, excluding these patients from the present analysis entirely may have led to even more biased estimations.

Overall, this secondary analysis demonstrates that older patients in Europe underwent outpatient surgery with a low risk of mortality. In comparison to inpatients, they presented with fewer comorbidities and higher functional and cognitive baseline status. They underwent more often minor procedures with minimal anaesthesia techniques and had better postoperative functional and cognitive scores. Further studies designed to assess functional and cognitive outcomes after outpatient surgery are needed to develop scores that facilitate the identification of older patients suited for outpatient surgery.

## AUTHOR CONTRIBUTIONS

**Linda Grüßer**: Methodology, investigation, data curation, validation, visualization, and wrote the original draft. **Mark Coburn**: Conceptualization, methodology, validation, supervision, project administration, writing—review and editing. **Matthias Schmid**: Methodology, software, formal analysis, visualization, writing—review and editing. **Rolf Rossaint**: Conceptualization, project administration, writing—review and editing. **Sebastian Ziemann**: Data curation, writing—review and editing. **Ana Kowark**: Conceptualization, methodology, data curation, validation, supervision, writing—review, and editing. **POSE Study group**: Data acquisition.

## FUNDING INFORMATION

The main study was supported by the European Society of Anaesthesiology and Intensive Care (ESAIC) as an ESAIC Research Group. MS received funding from the ESAIC POSE Research Group for this secondary analysis. The remaining authors report no funding for this secondary analysis.

## CONFLICT OF INTEREST STATEMENT

Outside the submitted work, LG reported payment for a lecture, Baxter Germany GmbH; MC reported payments for lectures, Baxter Germany GmbH, Drägerwerk AG & Co. KGaA, Ambu GmbH; RR reported being a cofounder of Docs in Clouds GmbH; AK reported being a member of the advisory board of PAION.

## Supporting information


**Supplemental Table 1.** Comorbidities and medication.

**Supplemental Table 2.** Study population.

**Supplemental Table 3.** Cross tabulations of functional status at baseline and follow‐up. (a) All patients, (b) outpatients, (c) inpatients.

**Supplemental Table 4.** Multivariable ordinal logistic regression—functional status.

**Supplemental Table 5.** Cross tabulations of cognitive status at baseline and follow‐up. (a) All patients, (b) outpatients, (c) inpatients.

**Supplemental Table 6.** Multivariable quasi‐Poisson regression—cognitive status.

**Supplemental Table 7.** Additional outcomes of outpatients and inpatients.

**Supplemental Table 8.** Baseline characteristics, perioperative characteristics and outcomes of planned outpatients with no and with unplanned stay.

**Data S1:** POSE‐study group.

## Data Availability

The data that supports the findings of this study are available in the supplementary material of this article.
