# Supplementary material for: Characteristics and outcomes of older patients undergoing out‐ versus inpatient surgery in Europe. A secondary analysis of the Peri‐interventional Outcome Study in the Elderly (POSE)
Source: Acta Anaesthesiol Scand. 2025 Mar 24;69(4):e70021. doi: 10.1111/aas.70021 (PMC11932067; doi:10.1111/aas.70021)
Supplement: Supplementary file 1 — Supplemental Table 1. Comorbidities and medication. [file AAS-69-0-s009.pdf]

Supplementary 1. Baseline comorbidities and medications of outpatients and inpatients

	Outpatients (*n*=1935)	Inpatients (*n*=7562)	Overall (*n*=9497)
**Diabetes**			
no	1572 (81.2%)	5978 (79.1%)	7550 (79.5%)
yes	363 (18.8%)	1584 (20.9%)	1947 (20.5%)
**Dyspnoea**			
no	1549 (80.1%)	5302 (70.1%)	6851 (72.1%)
with moderate exertion	353 (18.2%)	1958 (25.9%)	2311 (24.3%)
at rest	31 (1.6%)	283 (3.7%)	314 (3.3%)
missing	2 (0.1%)	19 (0.3%)	21 (0.2%)
**COPD**			
no	1747 (90.3%)	6789 (89.8%)	8536 (89.9%)
yes	188 (9.7%)	773 (10.2%)	961 (10.1%)
**History COPD**			
no	1782 (92.1%)	6975 (92.0%)	8739 (92.0%)
yes	153 (7.9%)	603 (8.0%)	756 (8.0%)
missing	0 (0%)	2 (0.0%)	2 (0.0%)
**Chronic respiratory failure**			
no	1871 (96.7%)	7236 (95.7%)	9107 (95.9%)
yes	64 (3.3%)	326 (4.3%)	390 (4.1%)
**Current smoker**			
no	1852 (95.7%)	7091 (93.8%)	8943 (94.2%)
yes	83 (4.3%)	457 (6.0%)	540 (5.7%)
missing	0 (0%)	14 (0.2%)	14 (0.1%)
**Acute renal failure**			
no	1905 (98.5%)	7246 (95.8%)	9151 (96.4%)
yes	30 (1.6%)	316 (4.2%)	346 (3.6%)
**Chronic renal failure**			
no	1690 (87.3%)	6134 (81.1%)	7824 (82.4%)

	Outpatients (*n*=1935)	Inpatients (*n*=7562)	Overall (*n*=9497)
yes	245 (12.7%)	1428 (18.9%)	1673 (17.6%)
**Dialysis**			
no	1908 (98.6%)	7455 (98.6%)	9363 (98.6%)
yes	27 (1.4%)	107 (1.4%)	134 (1.4%)
**Hypertension requiring medication**			
no	533 (27.5%)	1874 (24.8%)	2407 (25.3%)
yes	1402 (72.5%)	5688 (75.2%)	7090 (74.7%)
**Congestive heart failure**			
no	1771 (91.5%)	6269 (82.9%)	8040 (84.7%)
yes	163 (8.4%)	1290 (17.1%)	1453 (15.3%)
missing	1 (0.1%)	3 (0.0%)	4 (0.0%)
**Ischemic heart disease**			
no	1552 (80.2%)	5481 (72.5%)	7033 (74.1%)
yes	383 (19.8%)	2081 (27.5%)	2464 (25.9%)
**Cardiac arrhythmia**			
no	1448 (74.8%)	5049 (66.8%)	6497 (68.4%)
yes	487 (25.2%)	2513 (33.2%)	3000 (31.6%)
**Chronic heart failure**			
no	1620 (83.7%)	5768 (76.3%)	7388 (77.8%)
yes	315 (16.3%)	1794 (23.7%)	2109 (22.2%)
**Peripheral vascular disease**			
no	1797 (92.9%)	6644 (87.9%)	8441 (88.9%)
yes	138 (7.1%)	918 (12.1%)	1056 (11.1%)
**Hemiplegia**			
no	1905 (98.4%)	7361 (97.3%)	9266 (97.6%)
yes	30 (1.6%)	201 (2.7%)	231 (2.4%)
**Alcohol (number units/week)**			
Mean ± SD	1.38 ± 3.90	1.46 ± 4.64	1.44 ± 4.50
Median (IQR)	0 (0-0)	0 (0-0)	0 (0-0)

	Outpatients (*n*=1935)	Inpatients (*n*=7562)	Overall (*n*=9497)
Missing	32 (1.7%)	103 (1.4%)	135 (1.4%)
**Chronic alcohol abuse**			
no	1916 (99.0%)	7438 (98.4%)	9354 (98.5%)
yes	19 (1.0%)	124 (1.6%)	143 (1.5%)
**Cancer**			
no	1610 (83.2%)	5616 (74.3%)	7226 (76.1%)
yes	325 (16.8%)	1946 (25.7%)	2271 (23.9%)
**Disseminated cancer**			
no	1881 (97.2%)	7103 (93.9%)	8984 (94.6%)
yes	54 (2.8%)	456 (6.0%)	510 (5.4%)
missing	0 (0%)	3 (0.0%)	3 (0.0%)
**Transplanted organs**			
no	1933 (99.9%)	7552 (99.9%)	9485 (99.9%)
yes	2 (0.1%)	10 (0.1%)	12 (0.1%)
**Dementia**			
no	1837 (94.9%)	6904 (91.3%)	8741 (92.0%)
yes	98 (5.1%)	658 (8.7%)	756 (8.0%)
**Cerebrovascular disease**			
no	1751 (90.5%)	6500 (86.0%)	8251 (86.9%)
yes	184 (9.5%)	1062 (14.0%)	1246 (13.1%)
**Mild cognitive impairment**			
no	1803 (93.2%)	6617 (87.5%)	8420 (88.7%)
yes	132 (6.8%)	945 (13.8%)	1077 (11.3%)
**Other cognitive complaints**			
no	1660 (85.8%)	6515 (86.2%)	8175 (86.1%)
yes	275 (14.2%)	1047 (13.8%)	1322 (13.9%)
**Anticoagulants**			
no	1561 (80.7%)	5460 (72.2%)	7021 (73.9%)
yes	373 (19.3%)	2097 (27.7%)	2470 (26.0%)

	Outpatients (*n*=1935)	Inpatients (*n*=7562)	Overall (*n*=9497)
missing	1 (0.1%)	5 (0.1%)	6 (0.1%)
**Antiplatelet therapy**			
no	1276 (65.9%)	4634 (61.3%)	5910 (62.2%)
yes	658 (34.0%)	2924 (38.7%)	3582 (37.7%)
missing	1 (0.1%)	4 (0.1%)	5 (0.1%)
**Betablockers**			
no	1313 (67.9%)	4403 (58.2%)	5716 (60.2%)
yes	621 (32.1%)	3155 (41.7%)	3776 (39.8%)
Missing	1 (0.1%)	4 (0.1%)	5 (0.1%)
**ACE inhibitors**			
no	946 (48.9%)	3591 (47.5%)	4537 (47.8%)
yes	988 (51.1%)	3967 (52.5%)	4955 (52.2%)
missing	1 (0.1%)	4 (0.1%)	5 (0.1%)
**Antidepressants**			
no	1732 (89.5%)	6530 (86.4%)	8262 (87.0%)
yes	202 (10.4%)	1028 (13.6%)	1230 (13.0%)
missing	1 (0.1%)	4 (0.1%)	5 (0.1%)
**Neuroleptics**			
no	1866 (96.4%)	7140 (94.4%)	9006 (94.8%)
yes	68 (3.5%)	418 (5.5%)	486 (5.1%)
missing	1 (0.1%)	4 (0.1%)	5 (0.1%)
**Benzodiazepines**			
no	1702 (88.0%)	6577 (87.0%)	8279 (87.2%)
yes	232 (12.0%)	981 (13.0%)	1213 (12.8%)
missing	1 (0.1%)	4 (0.1%)	5 (0.1%)
**Z-drugs**			
no	1871 (96.7%)	7195 (95.1%)	9066 (95.5%)
yes	63 (3.3%)	363 (4.8%)	426 (4.5%)
missing	1 (0.1%)	4 (0.0%)	5 (0.1%)

Data are presented as n (%), mean ± SD or median (IQR).

Abbreviations: COPD=Chronic obstructive pulmonary disease; ACE=Angiotensin-converting enzyme
