# Supplementary material for: Characteristics and outcomes of older patients undergoing out‐ versus inpatient surgery in Europe. A secondary analysis of the Peri‐interventional Outcome Study in the Elderly (POSE)
Source: Acta Anaesthesiol Scand. 2025 Mar 24;69(4):e70021. doi: 10.1111/aas.70021 (PMC11932067; doi:10.1111/aas.70021)
Supplement: Supplementary file 2 — Supplemental Table 2. Study population. [file AAS-69-0-s005.pdf]

9497 patients in the POSE database
- 1935 outpatients
- 7562 inpatients

Functional Outcome

Patients with missing data at baseline or follow up were excluded from the analysis.

- 3 patients with missing data at baseline only
- 912 patients with missing data at follow-up only
- 3 patients with missing data at baseline and follow-up
- The functional status at follow-up of 386 patients who died, but had available data on functional status at baseline, was set to “totally dependent“ accounting for the expected missing data due to mortality.

↓

8965 patients included
in the functional outcome anaylsis
(9497-3-912-3+386=8965)

Cognitive Outcome

Patients with missing data at baseline or follow up were excluded from the analysis.

- 192 patients with missing data only at baseline
- 1465 patients with missing data only at follow-up
- 181 patients with missing data at baseline and follow-up
- The cognitive status at follow-up for 356 patients, who died, but had available data on the cognitive status at baseline, was set to “zero recalled words“ accounting for the expected missing data due to mortality

↓

8015 patients included
in the cognitive outcome anaylsis
(9497-192-1465-181+356=8015)

Time to death

For the time to death analysis the entire cohort was included (*POSE-Study group, Peri-interventional outcome study in the elderly in Europe. A 30-day prospective cohort study. Eur J Anaesthesiol, 2022; 39:198-209*)

↓

9497 patients included
in the time to death anaylsis
