# Supplementary material for: Characteristics and outcomes of older patients undergoing out‐ versus inpatient surgery in Europe. A secondary analysis of the Peri‐interventional Outcome Study in the Elderly (POSE)
Source: Acta Anaesthesiol Scand. 2025 Mar 24;69(4):e70021. doi: 10.1111/aas.70021 (PMC11932067; doi:10.1111/aas.70021)
Supplement: Supplementary file 3 — Supplemental Table 3. Cross tabulations of functional status at baseline and follow‐up. (a) All patients, (b) outpatients, (c) inpatients. [file AAS-69-0-s006.pdf]

Supplement 3 a-c. Functional status at baseline and follow-up.

a) All patients analyzed for the outcome functional status

Functional status baseline	*Functional status follow-up*			***Total***
	Independent	Partially dependent	Totally dependent	
Independent	3972<br>71.5 %<br>92.8 %<br>44.3 %	1253<br>22.5 %<br>40.5 %<br>14 %	333<br>6 %<br>21 %<br>3.7 %	5558<br>100 %<br>62 %<br>62 %
Partially dependent	295<br>10.8 %<br>6.9 %<br>3.3 %	1764<br>64.7 %<br>57 %<br>19.7 %	669<br>24.5 %<br>42.1 %<br>7.5 %	2728<br>100 %<br>30.4 %<br>30.5 %
Totally dependent	15<br>2.2 %<br>0.4 %<br>0.2 %	77<br>11.3 %<br>2.5 %<br>0.9 %	587<br>86.5 %<br>36.9 %<br>6.5 %	679<br>100 %<br>7.6 %<br>7.6 %
Total	4282<br>47.8 %<br>100 %<br>47.8 %	3094<br>34.5 %<br>100 %<br>34.5 %	1589<br>17.7 %<br>100 %<br>17.7 %	8965<br>100 %<br>100 %<br>100 %

b) All **outpatients** analyzed for the outcome functional status

*Functional status baseline**	*Functional status follow-up***			***Total***
	Independent	Partially dependent	Totally dependent	
Independent	1243<br>90.9 %<br>94 %<br>67.6 %	114<br>8.3 %<br>26.6 %<br>6.2 %	10<br>0.7 %<br>11.6 %<br>0.5 %	1367<br>100 %<br>74.4 %<br>74.3 %
Partially dependent	76<br>18.7 %<br>5.7 %<br>4.1 %	308<br>75.7 %<br>71.8 %<br>16.8 %	23<br>5.7 %<br>26.7 %<br>1.3 %	407<br>100 %<br>22.1 %<br>22.2 %
Totally dependent	4<br>6.2 %<br>0.3 %<br>0.2 %	7<br>10.9 %<br>1.6 %<br>0.4 %	53<br>82.8 %<br>61.6 %<br>2.9 %	64<br>100 %<br>3.5 %<br>3.5 %
Total	1323<br>72 %	429<br>23.3 %	86<br>4.7 %	1838<br>100 %

	100 %<br>72 %	100 %<br>23.3 %	100 %<br>4.7 %	100 %<br>100 %

c) All **inpatients** analyzed for the outcome functional status

Functional status baseline	*Functional status follow-up*			***Total***
	Independent	Partially dependent	Totally dependent	
Independent	2729<br>65.1 %<br>92.2 %<br>38.3 %	1139<br>27.2 %<br>42.7 %<br>16 %	323<br>7.7 %<br>21.5 %<br>4.5 %	4191<br>100 %<br>58.8 %<br>58.8 %
Partially dependent	219<br>9.4 %<br>7.4 %<br>3.1 %	1456<br>62.7 %<br>54.6 %<br>20.4 %	646<br>27.8 %<br>43 %<br>9.1 %	2321<br>100 %<br>32.6 %<br>32.6 %
Totally dependent	11<br>1.8 %<br>0.4 %<br>0.2 %	70<br>11.4 %<br>2.6 %<br>1 %	534<br>86.8 %<br>35.5 %<br>7.5 %	615<br>100 %<br>8.6 %<br>8.7 %
Total	2959<br>41.5 %<br>100 %<br>41.5 %	2665<br>37.4 %<br>100 %<br>37.4 %	1503<br>21.1 %<br>100 %<br>21.1 %	7127<br>100 %<br>100 %<br>100 %
