# Supplementary material for: Characteristics and outcomes of older patients undergoing out‐ versus inpatient surgery in Europe. A secondary analysis of the Peri‐interventional Outcome Study in the Elderly (POSE)
Source: Acta Anaesthesiol Scand. 2025 Mar 24;69(4):e70021. doi: 10.1111/aas.70021 (PMC11932067; doi:10.1111/aas.70021)
Supplement: Supplementary file 4 — Supplemental Table 4. Multivariable ordinal logistic regression—functional status. [file AAS-69-0-s004.pdf]

Supplement 4.

Functional status at baseline	Patient Status	Estimated probabilities of functional status at follow-up		
		Independent	Partially dependent	Totally dependent
Independent	outpatient	0.909	0.085	0.006
Independent	inpatient	0.633	0.327	0.04
Partially dependent	outpatient	0.281	0.557	0.162
Partially dependent	inpatient	0.14	0.544	0.316
Totally dependent	outpatient	0.015	0.174	0.811
Totally dependent	inpatient	0.01	0.124	0.866

The estimated probabilities of the functional status categories at follow-up obtained from multivariable ordinal logistic regression including 9 confounder variables, averaged across the 12 imputations
