# Supplementary material for: Characteristics and outcomes of older patients undergoing out‐ versus inpatient surgery in Europe. A secondary analysis of the Peri‐interventional Outcome Study in the Elderly (POSE)
Source: Acta Anaesthesiol Scand. 2025 Mar 24;69(4):e70021. doi: 10.1111/aas.70021 (PMC11932067; doi:10.1111/aas.70021)
Supplement: Supplementary file 5 — Supplemental Table 5. Cross tabulations of cognitive status at baseline and follow‐up. (a) All patients, (b) outpatients, (c) inpatients. [file AAS-69-0-s003.pdf]

Supplement 5. Cognitive status at baseline and follow-up.

a) All patients analyzed for the outcome cognitive status

Number of recalled words at baseline	*Number of recalled words at follow-up*				***Total***
	0	1	2	3	
0	913<br>65.6 %<br>48.7 %<br>11.4 %	138<br>9.9 %<br>16.2 %<br>1.7 %	176<br>12.6 %<br>9.6 %<br>2.2 %	165<br>11.9 %<br>4.8 %<br>2.1 %	1392<br>100 %<br>17.4 %<br>17.4 %
1	343<br>24.2 %<br>18.3 %<br>4.3 %	324<br>22.8 %<br>38.1 %<br>4 %	335<br>23.6 %<br>18.3 %<br>4.2 %	416<br>29.3 %<br>12 %<br>5.2 %	1418<br>100 %<br>17.7 %<br>17.7 %
2	323<br>14.9 %<br>17.2 %<br>4 %	221<br>10.2 %<br>26 %<br>2.8 %	724<br>33.5 %<br>39.5 %<br>9 %	895<br>41.4 %<br>25.9 %<br>11.2 %	2163<br>100 %<br>27 %<br>27 %
3	294<br>9.7 %<br>15.7 %<br>3.7 %	167<br>5.5 %<br>19.6 %<br>2.1 %	597<br>19.6 %<br>32.6 %<br>7.4 %	1984<br>65.2 %<br>57.3 %<br>24.8 %	3042<br>100 %<br>38 %<br>38 %
Total	1873<br>23.4 %<br>100 %<br>23.4 %	850<br>10.6 %<br>100 %<br>10.6 %	1832<br>22.9 %<br>100 %<br>22.9 %	3460<br>43.2 %<br>100 %<br>43.2 %	8015<br>100 %<br>100 %<br>100 %

b) All **outpatients** analyzed for the outcome cognitive status

Number of recalled words at baseline	*Number of recalled words at follow-up*				***Total***
	0	1	2	3	
0	100<br>50.3 %<br>45.5 %<br>6 %	30<br>15.6 %<br>17.8 %<br>1.8 %	26<br>13.1 %<br>6.3 %<br>1.6 %	43<br>21.6 %<br>5 %<br>2.6 %	199<br>100 %<br>12 %<br>12 %
1	49<br>17.8 %<br>22.3 %<br>2.9 %	64<br>23.2 %<br>37.9 %<br>3.8 %	75<br>27.2 %<br>18.1 %<br>4.5 %	88<br>31.9 %<br>10.2 %<br>5.3 %	276<br>100 %<br>16.6 %<br>16.5 %
2	39<br>8.3 %<br>17.7 %<br>2.3 %	42<br>9 %<br>24.9 %<br>2.5 %	168<br>35.8 %<br>40.6 %<br>10.1 %	220<br>46.9 %<br>25.6 %<br>13.2 %	469<br>100 %<br>28.2 %<br>28.1 %

3	32<br>4.4 %<br>14.5 %<br>1.9 %	33<br>4.6 %<br>19.5 %<br>2 %	145<br>20.1 %<br>35 %<br>8.7 %	510<br>70.8 %<br>59.2 %<br>30.6 %	720<br>100 %<br>43.3 %<br>43.2 %
Total	220<br>13.2 %<br>100 %<br>13.2 %	169<br>10.2 %<br>100 %<br>10.2 %	414<br>24.9 %<br>100 %<br>24.9 %	861<br>51.7 %<br>100 %<br>51.7 %	1664<br>100 %<br>100 %<br>100 %

c) All **inpatients** analyzed for the outcome cognitive status

Number of recalled words at baseline	*Number of recalled words at follow-up*				***Total***
	0	1	2	3	
0	813<br>68.1 %<br>49.2 %<br>12.8 %	108<br>9.1 %<br>15.9 %<br>1.7 %	150<br>12.6 %<br>10.6 %<br>2.4 %	122<br>10.2 %<br>4.7 %<br>1.9 %	1193<br>100 %<br>18.8 %<br>18.8 %
1	294<br>25.7 %<br>17.8 %<br>4.6 %	260<br>22.8 %<br>38.2 %<br>4.1 %	260<br>22.8 %<br>18.3 %<br>4.1 %	328<br>28.7 %<br>12.6 %<br>5.2 %	1142<br>100 %<br>18 %<br>18 %
2	284<br>16.8 %<br>17.2 %<br>4.5 %	179<br>10.6 %<br>26.3 %<br>2.8 %	556<br>32.8 %<br>39.2 %<br>8.8 %	675<br>39.8 %<br>26 %<br>10.6 %	1694<br>100 %<br>26.7 %<br>26.7 %
3	262<br>11.3 %<br>15.8 %<br>4.1 %	134<br>5.8 %<br>19.7 %<br>2.1 %	452<br>19.5 %<br>31.9 %<br>7.1 %	1474<br>63.4 %<br>56.7 %<br>23.2 %	2322<br>100 %<br>36.6 %<br>36.5 %
Total	1653<br>26 %<br>100 %<br>26 %	681<br>10.7 %<br>100 %<br>10.7 %	1418<br>22.3 %<br>100 %<br>22.3 %	2599<br>40.9 %<br>100 %<br>40.9 %	6351<br>100 %<br>100 %<br>100 %
