# Supplementary material for: Characteristics and outcomes of older patients undergoing out‐ versus inpatient surgery in Europe. A secondary analysis of the Peri‐interventional Outcome Study in the Elderly (POSE)
Source: Acta Anaesthesiol Scand. 2025 Mar 24;69(4):e70021. doi: 10.1111/aas.70021 (PMC11932067; doi:10.1111/aas.70021)
Supplement: Supplementary file 6 — Supplemental Table 6. Multivariable quasi‐Poisson regression—cognitive status. [file AAS-69-0-s007.pdf]

Supplement 6.

Number of recalled words at baseline	Patient Status	Estimated mean of the number of recalled words at follow-up
0	outpatient	1.04
0	inpatient	0.92
1	outpatient	1.87
1	inpatient	1.07
2	outpatient	2.61
2	inpatient	2.28
3	outpatient	2.7
3	inpatient	2.65

The estimated means of the number of recalled words at follow-up obtained from the multivariate ordinal logistic regression model including the 9 confounder variables averaged across the 12 imputations.
