# Supplementary material for: Characteristics and outcomes of older patients undergoing out‐ versus inpatient surgery in Europe. A secondary analysis of the Peri‐interventional Outcome Study in the Elderly (POSE)
Source: Acta Anaesthesiol Scand. 2025 Mar 24;69(4):e70021. doi: 10.1111/aas.70021 (PMC11932067; doi:10.1111/aas.70021)
Supplement: Supplementary file 7 — Supplemental Table 7. Additional outcomes of outpatients and inpatients. [file AAS-69-0-s001.pdf]

Supplementary 7. Additional outcomes of outpatients and inpatients

	Outpatient (*n*=1935)	Inpatient (*n*=7562)	Overall (*n*=9497)
**Discharge destination**			
Other hospital	16 (0.8%)	347 (4.6%)	363 (3.8%)
Rehabilitation	7 (0.4%)	931 (12.3%)	938 (9.9%)
Nursing home	69 (3.6%)	621 (8.2%)	690 (7.3%)
Home	1812 (93.6%)	4985 (65.9%)	6797 (71.6%)
Other	27 (1.4%)	73 (1.0%)	100 (1.1%)
Missing	4 (0.2%)	605 (8.0%)	609 (6.4%)
**In-hospital death before day 30**			
yes	2 (0.1%)	293 (3.9%)	295 (3.1%)
no	1933 (99.9%)	7269 (96.1%)	9202 (96.9%)
**Discharged before day 30**			
yes	1931 (99.8%)	6963 (92.1%)	8894 (93.7%)
no	2 (0.1%)	306 (4.0%)	308 (3.2%)
Missing	2 (0.1%)	293 (3.9%)	295 (3.1%)
***In-hospital* outcome according to the ACSNSQIP**			
- **Cardiac arrest**			
no	1932 (99.8%)	7343 (97.1%)	9275 (97.7%)
yes	3 (0.2%)	219 (2.9%)	222 (2.3%)
- **Myocardial infarction**			
no	1934 (99.9%)	7480 (98.9%)	9414 (99.1%)
yes	1 (0.1%)	82 (1.1%)	83 (0.9%)
- **Pneumonia**			
no	1933 (99.9%)	7229 (95.6%)	9162 (96.5%)
yes	2 (0.1%)	333 (4.4%)	335 (3.5%)
- **Pulmonary embolism**			
no	1935 (100%)	7525 (99.5%)	9460 (99.6%)
yes	0 (0%)	37 (0.5%)	37 (0.4%)

	Outpatient (*n*=1935)	Inpatient (*n*=7562)	Overall (*n*=9497)
- **Unplanned intubation**			
no	1935 (100%)	7488 (99.0%)	9423 (99.2%)
yes	0 (0%)	74 (1.0%)	74 (0.8%)
- **Ventilator 48h**			
no	1935 (100%)	7414 (98.0%)	9349 (98.4%)
yes	0 (0%)	148 (2.0%)	148 (1.6%)
- **Return to the operating room**			
no	1925 (99.5%)	7203 (95.3%)	9128 (96.1%)
yes	10 (0.5%)	359 (4.7%)	369 (3.9%)
- **Stroke**			
no	1935 (100%)	7510 (99.3%)	9445 (99.5%)
yes	0 (0%)	52 (0.7%)	52 (0.5%)
- **Acute kidney injury**			
no	1930 (99.7%)	7250 (95.9%)	9180 (96.7%)
yes	5 (0.3%)	312 (4.1%)	317 (3.3%)
- **Deep vein thrombosis**			
no	1935 (100%)	7529 (99.6%)	9464 (99.7%)
yes	0 (0%)	33 (0.4%)	33 (0.3%)
- **Venous thromboembolism requiring therapy**			
no	1935 (100%)	7535 (99.6%)	9470 (99.7%)
yes	0 (0%)	27 (0.4%)	27 (0.3%)
- **Superficial incisional surgical site infection**			
no	1926 (99.5%)	7404 (97.9%)	9330 (98.2%)
yes	9 (0.5%)	158 (2.1%)	167 (1.8%)
- **Deep incisional surgical site infection**			
no	1935 (100%)	7430 (98.3%)	9365 (98.6%)
yes	0 (0%)	132 (1.7%)	132 (1.4%)

	Outpatient (*n*=1935)	Inpatient (*n*=7562)	Overall (*n*=9497)
- **Organ space surgical site infection**			
no	1935 (100%)	7514 (99.4%)	9449 (99.5%)
yes	0 (0%)	48 (0.6%)	48 (0.5%)
- **Wound disruption**			
no	1935 (100%)	7438 (98.4%)	9373 (98.7%)
yes	0 (0%)	124 (1.6%)	124 (1.3%)
- **Systemic sepsis**			
no	1932 (99.8%)	7354 (97.2%)	9286 (97.8%)
yes	3 (0.2%)	208 (2.8%)	211 (2.2%)
- **Urinary tract infection**			
no	1920 (99.2%)	7238 (95.7%)	9158 (96.4%)
yes	15 (0.8%)	324 (4.3%)	339 (3.6%)
- **Discharge to post-acute care**			
no	1840 (95.1%)	5630 (74.5%)	7470 (78.7%)
yes	95 (4.9%)	1932 (25.5%)	2027 (21.3%)
**Telephone follow-up status day 30:**			
Alive	1840 (95.1%)	6546 (86.6%)	8386 (88.3%)
Dead	4 (0.2%)	89 (1.2%)	93 (1.0%)
Follow-up not performed	87 (4.5%)	328 (4.3%)	415 (4.4%)
Missing	4 (0.2%)	599 (7.9%)	603 (6.3%)
**Telephone follow-up day 30: Any complications after discharge**			
yes	16 (0.8%)	319 (4.2%)	335 (3.5%)
- Cardiac	3 (0.2%)	103 (1.4%)	106 (1.1%)
- Pulmonary	8 (0.4%)	144 (1.9%)	152 (1.6%)
- Stroke	1 (0.1%)	33 (0.4%)	34 (0.4%)
- Acute kidney injury	5 (0.3%)	94 (1.2%)	99 (1.0%)
no	1871 (96.7%)	6513 (86.1%)	8384 (88.3%)
Missing	48 (2.5%)	730 (9.7%)	778 (8.2%)

Data are presented as n (%).
