# Supplementary material for: Characteristics and outcomes of older patients undergoing out‐ versus inpatient surgery in Europe. A secondary analysis of the Peri‐interventional Outcome Study in the Elderly (POSE)
Source: Acta Anaesthesiol Scand. 2025 Mar 24;69(4):e70021. doi: 10.1111/aas.70021 (PMC11932067; doi:10.1111/aas.70021)
Supplement: Supplementary file 8 — Supplemental Table 8. Baseline characteristics, perioperative characteristics and outcomes of planned outpatients with no and with unplanned stay. [file AAS-69-0-s002.pdf]

Supplementary 8a. Baseline characteristics of planned outpatients with no overnight stay as planned (Outpatients = 0 days) and planned outpatients with an unplanned inpatient stay (Outpatients > 0 days)

	**Outpatients = 0 days (*n*=1834)**	**Outpatients > 0 days (*n*=101)**	**Overall planned outpatients (*n*=1935)**
**Age (y)**	83.0 (81.0-86.0)	84.0 (81.0-86.0)	83.0 (81.0-86.0)
**Sex**			
male	861 (46.9%)	50 (49.5%)	911 (47.1%)
female	973 (53.1%)	51 (50.5%)	1024 (52.9%)
**Height (cm)**	164 (157-170)	165 (158-170.0)	164 (157.0-170.0)
Missing	14 (0.8%)	26 (25.7%)	40 (2.1%)
**Weight (kg)**	70.0 (61.0-79.0)	68.5 (60.0-78.0)	70.00 (61.0-79.0)
Missing	10 (0.5%)	17 (16.8%)	27 (1.4%)
**ASA score**			
1	70 (3.8%)	4 (4.0%)	74 (3.8%)
2	972 (53.0%)	51 (50.5%)	1023 (52.9%)
3	765 (41.7%)	43 (42.6%)	808 (41.8%)
4	26 (1.4%)	3 (3.0%)	29 (1.5%)
5	0 (0%)	0 (0%)	0 (0%)
Missing	1 (0.1%)	0 (0%)	1 (0.1%)
**Referring facility**			
home	1672 (91.2%)	93 (92.1%)	1765 (91.2%)
other hospital	7 (0.4%)	1 (1.0%)	8 (0.4%)
rehabilitation	2 (0.1%)	1 (1.0%)	3 (0.2%)

	Outpatients = 0 days (*n*=1834)	Outpatients > 0 days (*n*=101)	Overall planned outpatients (*n*=1935)
nursing home	69 (3.8%)	5 (5.0%)	74 (3.8%)
other	84 (4.6%)	1 (1.0%)	85 (4.4%)
**Multimorbidity[a]**	1249 (68.1%)	70 (69.3%)	1319 (68.2%)
**Haemoglobin (g dl^{-1})**	13.1 (11.8-14.2)	12.7 (11.0-14.2)	13.1 (11.8-14.2)
Missing	1074 (58.6%)	42 (41.6%)	1116 (57.7%)
**History of falls during the last 6 months**			
none	1486 (81.0%)	81 (80.2%)	6426 1567 (81.0%)
1 time	232 (12.7%)	12 (11.9%)	244 (12.6%)
>1 time	97 (5.3%)	8 (7.9%)	105 (5.4%)
Missing	19 (1.0%)	0 (0%)	19 (1.0%)
**Limited timed up& go test[b]**	1001 (54.6%)	73 (72.3%)	1074 (55.5%)
Missing	291 (15.9%)	14 (13.9%)	305 (15.8%)
**Unintentional weight loss ≥ 4.5 kg in the last year**	220 (12.0%)	18 (17.8%)	238 (12.3%)
Missing	22 (1.2%)	0 (0%)	22 (1.1%)
**Functional status at baseline**			
Independent	1365 (74.4%)	66 (65.3%)	1431 (74.0%)
Partially dependent	399 (21.8%)	31 (30.7%)	430 (22.2%)
Totally dependent	69 (3.8%)	4 (4.0%)	73 (3.8%)
Missing	1 (0.1%)	0 (0%)	1 (0.1%)

	Outpatients = 0 days (*n*=1834)	Outpatients > 0 days (*n*=101)	Overall planned outpatients (*n*=1935)
**Mini-Cog[c]: Total points**			
0	166 (9.1%)	14 (13.9%)	180 (9.3%)
1-3	769 (41.9%)	43 (42.6%)	812 (42.0%)
4-5	791 (43.1%)	39 (38.6%)	830 (42.9%)
Missing	108 (5.9%)	5 (5.0%)	113 (5.8%)
**Mini-Cog - mean number of correctly recalled words**	2.02 ± 1.04	1.82 ± 1.08	2.01 ± 1.05
Missing	65 (3.5%)	5 (5.0%)	70 (3.6%)
**Mini-Cog – mean number of clock draw points**	1.11 ± 0.994	0.979 ± 1.01	1.11 ± 0.995
Missing	108 (5.9%)	5 (5.0%)	113 (5.8%)

Data are presented as n (%), mean ± SD or median (IQR).

[a] Multimorbidity was defined as the presence of at least two of the assessed comorbidities.

[b] Timed up and go test was defined as limited when performed in >12s.

[c] Mini-Cog screening tool to detect cognitive impairment or dementia: 0=profound cognitive dysfunction, ≤3 cognitive impairment according to Robinson et al.*, 5=normal cognition.

* Robinson TN, Eiseman B, Wallace JI, et al. Redefining geriatric preoperative assessment using frailty, disability and co-morbidity. *Ann Surg*. Sep 2009;250(3):449-55.

Supplementary 8b. Perioperative characteristics of planned outpatients with no overnight stay as planned (Outpatients = 0 days) and planned outpatients with an unplanned inpatient stay (Outpatients > 0 days)

	Outpatients = 0 days (*n*=1834)	Outpatients > 0 days (*n*=101)	Overall planned outpatients (*n*=1935)
**Urgency**			
elective	1796 (97.9%)	95 (94.1%)	1891 (97.7%)
urgent	31 (1.7%)	2 (2.0%)	33 (1.7%)
emergency	7 (0.4%)	4 (4.0%)	11 (0.6%)
**Severity of surgery**			
minor	841 (45.9%)	54 (53.5%)	895 (46.3%)
intermediate	960 (52.3%)	31 (30.7%)	991 (51.2%)
major	33 (1.8%)	16 (15.8%)	49 (2.5%)
**Type of intervention**			
Abdominal	56 (3.1%)	6 (5.9%)	62 (3.2%)
Cardiovascular and thoracic	45 (2.5%)	2 (2.0%)	47 (2.4%)
ENT and ophthalmic	934 (50.9%)	36 (35.6%)	970 (50.1%)
Gynaecologic and urological	189 (10.3%)	29 (28.7%)	218 (11.3%)
Interventional	314 (17.1%)	13 (12.9%)	327 (16.9%)
Neurosurgery	2 (0.1%)	1 (1.0%)	3 (0.2%)
Orthopaedic, trauma and plastic	221 (12.1%)	12 (11.9%)	233 (12.0%)
Other surgery	73 (4.0%)	2 (2.0%)	75 (3.9%)

	Outpatients = 0 days (*n*=1834)	Outpatients > 0 days (*n*=101)	Overall planned outpatients (*n*=1935)
**Laparoscopic surgery**	23 (1.3%)	5 (5.0%)	28 (1.4%)
**Cancer surgery**	110 (6.0%)	22 (21.8%)	132 (6.8%)
**Median anaesthesia duration (min)**	32.0 (22.0-52.0)	50 (31.0-81.0)	33.0 (23.0-55.0)
**Anaesthesia technique**			
general	394 (21.5%)	44 (43.6%)	438 (22.6%)
regional[a]	252 (13.7%)	37 (36.6%)	289 (14.9%)
sedation	1006 (54.9%)	15 (14.9%)	1021 (52.8%)
combination[b]	182 (9.9%)	5 (5.0%)	187 (9.7%)
**Premedication before the intervention[c]**	455 (24.8%)	8 (7.9%)	463 (23.9%)
Missing	1 (0.1%)	0 (0%)	1 (0.1%)
**Application of safe surgery checklist**	1312 (71.5%)	85 (84.2%)	1397 (72.2%)
Missing	5 (0.3%)	0 (0%)	5 (0.3%)
**Transfusion of platelets**	0 (0%)	0 (0%)	0 (0%)
**Transfusion of plasma**	0 (0%)	0 (0%)	0 (0%)
**Transfusion of RBC**	1 (0.1%)	1 (1.0%)	2 (0.1%)
**ICU admission**	7 (0.4%)	4 (4.0%)	11 (0.6%)
**Admission to geriatric support unit**	15 (0.8%)	3 (3.0%)	18 (0.9%)
**Mean hospital length of stay (days)**	0 ± 0	3.32 ± 6.24	0.173 ± 1.60

	Outpatients = 0 days (*n*=1834)	Outpatients > 0 days (*n*=101)	Overall planned outpatients (*n*=1935)
**Median hospital length of stay (days)**	0 (0-0)	1 (1-2)	0 (0-0)

Data are presented as n (%), mean ± SD or median (IQR).

Abbreviations: ENT=ears, nose and throat; ICU=intensive care unit; RBC=red blood cells

[a]Regional anaesthesia includes epidural, spinal or other regional anaesthesia technique

[b]Combined anaesthesia is defined as a combination of at least two of the three given categories: general anaesthesia, sedation, or regional anaesthesia

[c]Premedication comprises benzodiazepine and clonidine

Supplementary 8c. Baseline comorbidities and medications of planned outpatients with no overnight stay as planned (outpatients = 0 days) and planned outpatients with an unplanned inpatient stay (outpatients > 0 days)

	**Outpatients = 0 days (*n*=1834)**	**Outpatients > 0 days (*n*=101)**	**Overall planned outpatients (*n*=1935)**
**Diabetes**			
no	1482 (80.8%)	90 (89.1%)	1572 (81.2%)
yes	352 (19.2%)	11 (10.9%)	363 (18.8%)
**Dyspnoea**			
no	1469 (80.1%)	80 (79.2%)	1549 (80.1%)
with moderate exertion	334 (18.2%)	19 (18.8%)	353 (18.2%)
at rest	29 (1.6%)	2 (2.0%)	31 (1.6%)
missing	2 (0.1%)	0 (0%)	2 (0.1%)
**COPD**			
no	1663 (90.7%)	84 (83.2%)	1747 (90.3%)
yes	171 (9.3%)	17 (16.8%)	188 (9.7%)
**History COPD**			
no	1693 (92.3%)	89 (88.1%)	1782 (92.1%)
yes	141 (7.7%)	12 (11.9%)	153 (7.9%)
**Chronic respiratory failure**			
no	1772 (96.6%)	99 (98.0%)	1871 (96.7%)
yes	62 (3.4%)	2 (2.0%)	64 (3.3%)
**Current smoker**			
no	1762 (96.1%)	90 (89.1%)	1852 (95.7%)
yes	72 (3.9%)	11 (10.9%)	83 (4.3%)
**Acute renal failure**			
no	1807 (98.5%)	98 (97.0%)	1905 (98.4%)
yes	27 (1.5%)	3 (3.0%)	30 (1.6%)

	Outpatients = 0 days (*n*=1834)	Outpatients > 0 days (*n*=101)	Overall planned outpatients (*n*=1935)
**Chronic renal failure**			
no	1602 (87.4%)	88 (87.1%)	1690 (87.3%)
yes	232 (12.7%)	13 (12.9%)	245 (12.7%)
**Dialysis**			
no	1807 (98.5%)	101 (100%)	1908 (98.6%)
yes	27 (1.5%)	0 (0%)	27 (1.4%)
**Hypertension requiring medication**			
no	505 (27.5%)	28 (27.7%)	533 (27.5%)
yes	1329 (72.5%)	73 (72.3%)	1402 (72.5%)
**Congestive heart failure**			
no	1682 (91.7%)	89 (88.1%)	1771 (91.5%)
yes	151 (8.2%)	12 (11.9%)	163 (8.4%)
missing	1 (0.1%)	0 (0%)	1 (0.1%)
**Ischemic heart disease**			
no	1471 (80.2%)	81 (80.2%)	1552 (80.2%)
yes	363 (19.8%)	20 (19.8%)	383 (19.8%)
**Cardiac arrhythmia**			
no	1373 (74.9%)	75 (74.3%)	1448 (74.8%)
yes	461 (25.1%)	26 (25.7%)	487 (25.2%)
**Chronic heart failure**			
no	1533 (83.6%)	87 (86.1%)	1620 (83.7%)
yes	301 (16.4%)	14 (13.9%)	315 (16.3%)
**Peripheral vascular disease**			
no	1703 (92.9%)	94 (93.1%)	1797 (92.9%)
yes	131 (7.1%)	7 (6.9%)	138 (7.1%)

	Outpatients = 0 days (*n*=1834)	Outpatients > 0 days (*n*=101)	Overall planned outpatients (*n*=1935)
**Hemiplegia**			
no	1805 (98.4%)	100 (99.0%)	1905 (98.4%)
yes	29 (1.6%)	1 (1.0%)	30 (1.6%)
**Alcohol (number units/week)**			
Mean (SD)	1.33 (3.84)	2.22 (4.89)	1.38 (3.90)
Median (IQR)	0 [0, 0]	0 [0, 1.50]	0 [0, 0]
Missing	30 (1.6%)	2 (2.0%)	32 (1.7%)
**Chronic alcohol abuse**			
no	1816 (99.0%)	100 (99.0%)	1916 (99.0%)
yes	18 (1.0%)	1 (1.0%)	19 (1.0%)
**Cancer**			
no	1537 (83.8%)	73 (72.3%)	1610 (83.2%)
yes	297 (16.2%)	28 (27.7%)	325 (16.8%)
**Disseminated cancer**			
no	1785 (97.3%)	96 (95.0%)	1881 (97.2%)
yes	49 (2.7%)	5 (5.0%)	54 (2.8%)
**Transplanted organs**			
no	1832 (99.9%)	101 (100%)	1933 (99.9%)
yes	2 (0.1%)	0 (0%)	2 (0.1%)
**Dementia**			
no	1746 (95.2%)	91 (90.1%)	1837 (94.9%)
yes	88 (4.8%)	10 (9.9%)	98 (5.1%)
**Cerebrovascular disease**			
no	1662 (90.6%)	89 (88.1%)	1751 (90.5%)
yes	172 (9.4%)	12 (11.9%)	184 (9.5%)

	**Outpatients = 0 days (*n*=1834)**	**Outpatients > 0 days (*n*=101)**	**Overall planned outpatients (*n*=1935)**
**Mild cognitive impairment**			
no	1714 (93.5%)	89 (88.1%)	1803 (93.2%)
yes	120 (6.5%)	12 (11.9%)	132 (6.8%)
**Other cognitive complaints**			
no	1566 (85.4%)	94 (93.1%)	1660 (85.8%)
yes	268 (14.6%)	7 (6.9%)	275 (14.2%)
**Anticoagulants**			
no	1478 (80.6%)	83 (82.2%)	1561 (80.7%)
yes	355 (19.4%)	18 (17.8%)	373 (19.3%)
missing	1 (0.1%)	0 (0%)	1 (0.1%)
**Antiplatelet therapy**			
no	1214 (66.2%)	62 (61.4%)	1276 (65.9%)
yes	619 (33.8%)	39 (38.6%)	658 (34.0%)
missing	1 (0.1%)	0 (0%)	1 (0.1%)
**Betablockers**			
no	1245 (67.9%)	68 (67.3%)	1313 (67.9%)
yes	588 (32.1%)	33 (32.7%)	621 (32.1%)
Missing	1 (0.1%)	0 (0%)	1 (0.1%)
**ACE inhibitors**			
no	885 (48.3%)	61 (60.4%)	946 (48.9%)
yes	948 (51.7%)	40 (39.6%)	988 (51.1%)
missing	1 (0.1%)	0 (0%)	1 (0.1%)
**Antidepressants**			
no	1641 (89.5%)	91 (90.1%)	1732 (89.5%)
yes	192 (10.5%)	10 (9.9%)	202 (10.4%)

	Outpatients = 0 days (*n*=1834)	Outpatients > 0 days (*n*=101)	Overall planned outpatients (*n*=1935)
missing	1 (0.1%)	0 (0%)	1 (0.1%)
**Neuroleptics**			
no	1767 (96.3%)	99 (98.0%)	1866 (96.4%)
yes	66 (3.6%)	2 (2.0%)	68 (3.5%)
missing	1 (0.1%)	0 (0%)	1 (0.1%)
**Benzodiazepines**			
no	220 (12.0%)	12 (11.9%)	232 (12.0%)
yes	1613 (87.9%)	89 (88.1%)	1702 (88.0%)
missing	1 (0.1%)	0 (0%)	1 (0.1%)
**Z-drugs**			
no	1775 (96.8%)	96 (95.0%)	1871 (96.7%)
yes	58 (3.2%)	5 (5.0%)	63 (3.3%)
missing	1 (0.1%)	0 (0%)	1 (0.1%)

Data are presented as n (%).

Abbreviations: COPD=Chronic obstructive pulmonary disease; ACE=Angiotensin-converting enzyme

Supplementary 8d. Functional outcomes of planned outpatients with no overnight stay as planned (Outpatients = 0 days) and planned outpatients with an unplanned inpatient stay (Outpatients > 0 days)

a) All **outpatients** analyzed for the outcome functional status

*Functional status baseline**	*Functional status follow-up***			***Total***
	Independent	Partially dependent	Totally dependent	
Independent	1243<br>90.9 %<br>94 %<br>67.6 %	114<br>8.3 %<br>26.6 %<br>6.2 %	10<br>0.7 %<br>11.6 %<br>0.5 %	1367<br>100 %<br>74.4 %<br>74.3 %
Partially dependent	76<br>18.7 %<br>5.7 %<br>4.1 %	308<br>75.7 %<br>71.8 %<br>16.8 %	23<br>5.7 %<br>26.7 %<br>1.3 %	407<br>100 %<br>22.1 %<br>22.2 %
Totally dependent	4<br>6.2 %<br>0.3 %<br>0.2 %	7<br>10.9 %<br>1.6 %<br>0.4 %	53<br>82.8 %<br>61.6 %<br>2.9 %	64<br>100 %<br>3.5 %<br>3.5 %
Total	1323<br>72 %<br>100 %<br>72 %	429<br>23.3 %<br>100 %<br>23.3 %	86<br>4.7 %<br>100 %<br>4.7 %	1838<br>100 %<br>100 %<br>100 %

b) All **planned outpatients with no overnight stay as planned (outpatients = 0 days of hospital stay)** analyzed for the outcome functional status

Functional status baseline	*Functional status follow-up*			***Total***
	Independent	Partially dependent	Totally dependent	
Independent	1192<br>91.5 %<br>94.5 %<br>68.5 %	103<br>7.9 %<br>25.6 %<br>5.9 %	8<br>0.6 %<br>10.8 %<br>0.5 %	1303<br>100 %<br>74.9 %<br>74.9 %
Partially dependent	66<br>17.6 %<br>5.2 %<br>3.8 %	293<br>77.9 %<br>72.7 %<br>16.8 %	17<br>4.5 %<br>23 %<br>1 %	376<br>100 %<br>21.6 %<br>21.6 %
Totally dependent	4<br>6.7 %	7<br>11.7 %	49<br>81.7 %	60<br>100 %

	0.3 %<br>0.2 %	1.7 %<br>0.4 %	66.2 %<br>2.8 %	3.5 %<br>3.4 %
Total	1262<br>72.6 %<br>100 %<br>72.6 %	403<br>23.2 %<br>100 %<br>23.2 %	74<br>4.3 %<br>100 %<br>4.3 %	1739<br>100 %<br>100 %<br>100 %

c) All **planned outpatients with an unplanned inpatient stay (outpatients > 0 days of hospital stay)** analyzed for the outcome functional status

Functional status baseline	*Functional status follow-up*			***Total***
	Independent	Partially dependent	Totally dependent	
Independent	51<br>79.7 %<br>83.6 %<br>51.5 %	11<br>17.2 %<br>42.3 %<br>11.1 %	2<br>3.1 %<br>16.7 %<br>2 %	64<br>100 %<br>64.6 %<br>64.6 %
Partially dependent	10<br>32.3 %<br>16.4 %<br>10.1 %	15<br>48.4 %<br>57.7 %<br>15.2 %	6<br>19.4 %<br>50 %<br>6.1 %	31<br>100 %<br>31.3 %<br>31.4 %
Totally dependent	0<br>0 %<br>0 %<br>0 %	0<br>0 %<br>0 %<br>0 %	4<br>100 %<br>33.3 %<br>4 %	4<br>100 %<br>4 %<br>4 %
Total	61<br>61.6 %<br>100 %<br>61.6 %	26<br>26.3 %<br>100 %<br>26.3 %	12<br>12.1 %<br>100 %<br>12.1 %	99<br>100 %<br>100 %<br>100 %

Supplementary 8e. Cognitive outcomes of planned outpatients with no overnight stay as planned (outpatients = 0 days) and planned outpatients with an unplanned inpatient stay (outpatients > 0 days)

a) All **outpatients** analyzed for the outcome cognitive status

Number of recalled words at baseline	*Number of recalled words at follow-up*				***Total***
	0	1	2	3	
0	100<br>50.3 %<br>45.5 %<br>6 %	30<br>15.6 %<br>17.8 %<br>1.8 %	26<br>13.1 %<br>6.3 %<br>1.6 %	43<br>21.6 %<br>5 %<br>2.6 %	199<br>100 %<br>12 %<br>12 %
1	49<br>17.8 %<br>22.3 %<br>2.9 %	64<br>23.2 %<br>37.9 %<br>3.8 %	75<br>27.2 %<br>18.1 %<br>4.5 %	88<br>31.9 %<br>10.2 %<br>5.3 %	276<br>100 %<br>16.6 %<br>16.5 %
2	39<br>8.3 %<br>17.7 %<br>2.3 %	42<br>9 %<br>24.9 %<br>2.5 %	168<br>35.8 %<br>40.6 %<br>10.1 %	220<br>46.9 %<br>25.6 %<br>13.2 %	469<br>100 %<br>28.2 %<br>28.1 %
3	32<br>4.4 %<br>14.5 %<br>1.9 %	33<br>4.6 %<br>19.5 %<br>2 %	145<br>20.1 %<br>35 %<br>8.7 %	510<br>70.8 %<br>59.2 %<br>30.6 %	720<br>100 %<br>43.3 %<br>43.2 %
Total	220<br>13.2 %<br>100 %<br>13.2 %	169<br>10.2 %<br>100 %<br>10.2 %	414<br>24.9 %<br>100 %<br>24.9 %	861<br>51.7 %<br>100 %<br>51.7 %	1664<br>100 %<br>100 %<br>100 %

b) All **planned outpatients with no overnight stay as planned (outpatients = 0 days of hospital stay)** analyzed for the outcome cognitive status

Number of recalled words at baseline	*Number of recalled words at follow-up*				***Total***
	0	1	2	3	
0	94<br>50.5 %<br>46.8 %<br>5.9 %	28<br>15.1 %<br>17.1 %<br>1.8 %	26<br>14 %<br>6.5 %<br>1.6 %	38<br>20.4 %<br>4.6 %<br>2.4 %	186<br>100 %<br>11.8 %<br>11.7 %
1	47<br>17.7 %	64<br>24.2 %	71<br>26.8 %	83<br>31.3 %	265<br>100 %

	23.4 %<br>3 %	39 %<br>4 %	17.8 %<br>4.5 %	10.1 %<br>5.2 %	16.8 %<br>16.7 %
2	32<br>7.3 %<br>15.9 %<br>2 %	40<br>9.1 %<br>24.4 %<br>2.5 %	162<br>36.9 %<br>40.7 %<br>10.2 %	205<br>46.7 %<br>25 %<br>13 %	439<br>100 %<br>27.7 %<br>27.7 %
3	28<br>4 %<br>13.9 %<br>1.8 %	32<br>4.6 %<br>19.5 %<br>2 %	139<br>20.1 %<br>34.9 %<br>8.8 %	493<br>71.2 %<br>60.2 %<br>31.2 %	692<br>100 %<br>43.7 %<br>43.8 %
Total	201<br>12.7 %<br>100 %<br>12.7 %	164<br>10.4 %<br>100 %<br>10.4 %	398<br>25.2 %<br>100 %<br>25.2 %	819<br>51.8 %<br>100 %<br>51.8 %	1582<br>100 %<br>100 %<br>100 %

c) All **planned outpatients with an unplanned inpatient stay (outpatients > 0 days of hospital stay)** analyzed for the outcome cognitive status

Number of recalled words at baseline	*Number of recalled words at follow-up*				***Total***
	0	1	2	3	
0	6<br>46.2 %<br>31.6 %<br>7.3 %	2<br>15.4 %<br>40 %<br>2.4 %	0<br>0 %<br>0 %<br>0 %	5<br>38.5 %<br>11.9 %<br>6.1 %	13<br>100 %<br>15.9 %<br>15.8 %
1	2<br>18.2 %<br>10.5 %<br>2.4 %	0<br>0 %<br>0 %<br>0 %	4<br>36.4 %<br>25 %<br>4.9 %	5<br>45.5 %<br>11.9 %<br>6.1 %	11<br>100 %<br>13.4 %<br>13.4 %
2	7<br>23.3 %<br>36.8 %<br>8.5 %	2<br>6.7 %<br>40 %<br>2.4 %	6<br>20 %<br>37.5 %<br>7.3 %	15<br>50 %<br>35.7 %<br>18.3 %	30<br>100 %<br>36.6 %<br>36.5 %
3	4<br>14.3 %<br>21.1 %<br>4.9 %	1<br>3.6 %<br>20 %<br>1.2 %	6<br>21.4 %<br>37.5 %<br>7.3 %	17<br>60.7 %<br>40.5 %<br>20.7 %	28<br>100 %<br>34.1 %<br>34.1 %
Total	19<br>23.2 %<br>100 %<br>23.2 %	5<br>6.1 %<br>100 %<br>6.1 %	16<br>19.5 %<br>100 %<br>19.5 %	42<br>51.2 %<br>100 %<br>51.2 %	82<br>100 %<br>100 %<br>100 %

Supplementary 8f. Additional outcomes of planned outpatients with no overnight stay as planned (outpatients = 0 days) and planned outpatients with an unplanned inpatient stay (outpatients > 0 days)

Survival

Log-rank test für Survival: p = 0.002

30-day survival probability all outpatients: 0.997 [0.994-0.999]

30-day survival probability of planned outpatients with no overnight stay as planned (outpatients = 0 days): 0.998 [0.996-1.000]

30-day survival probability planned outpatients with an unplanned inpatient stay (outpatients > 0 days): 0.980 [0.952-1.000]

Further outcomes

	**Outpatients = 0 days (*n*=1834)**	**Outpatients > 0 days (*n*=101)**	**Overall planned outpatients (*n*=1935)**
**Discharge destination**			
Other hospital	12 (0.7%)	4 (4.0%)	16 (0.8%)
Rehabilitation	5 (0.3%)	2 (2.0%)	7 (0.4%)
Nursing home	64 (3.5%)	5 (5.0%)	69 (3.6%)
Home	1726 (94.1%)	86 (85.1%)	1812 (93.6%)
Other	27 (1.5%)	0 (0%)	27 (1.4%)
Missing	0 (0%)	4 (4.0%)	4 (0.2%)
**In-hospital death before day 30**			
yes	0 (0%)	2 (2.0%)	2 (0.1%)
no	1834 (100%)	99 (98.0%)	1933 (99.9%)
**Discharged before day 30**			
yes	1834 (100%)	97 (96.0%)	1931 (99.8%)
no	0 (0%)	2 (2.0%)	2 (0.1%)
Missing	0 (0%)	2 (2.0%)	2 (0.1%)

	Outpatients = 0 days (*n*=1834)	Outpatients > 0 days (*n*=101)	Overall planned outpatients (*n*=1935)
***In-hospital* outcome according to the ACSNSQIP**			
- **Cardiac arrest**			
no	1833 (99.9%)	99 (98.0%)	1932 (99.8%)
yes	1 (0.1%)	2 (2.0%)	3 (0.2%)
- **Myocardial infarction**			
no	1834 (100%)	100 (99.0%)	1934 (99.9%)
yes	0 (0%)	1 (1.0%)	1 (0.1%)
- **Pneumonia**			
no	1833 (99.9%)	100 (99.0%)	1933 (99.9%)
yes	1 (0.1%)	1 (1.0%)	2 (0.1%)
- **Pulmonary embolism**			
no	1834 (100%)	101 (100%)	1935 (100%)
yes	0 (0%)	0 (0%)	0 (0%)
- **Unplanned intubation**			
no	1834 (100%)	101 (100%)	1935 (100%)
yes	0 (0%)	0 (0%)	0 (0%)
- **Ventilator 48h**			
no	1834 (100%)	101 (100%)	1935 (100%)
yes	0 (0%)	0 (0%)	0 (0%)
- **Return to the operating room**			
no	1827 (99.6%)	98 (97.0%)	1925 (99.5%)
yes	7 (0.4%)	3 (3.0%)	10 (0.5%)
- **Stroke**			
no	1834 (100%)	101 (100%)	1935 (100%)
yes	0 (0%)	0 (0%)	0 (0%)

	Outpatients = 0 days (*n*=1834)	Outpatients > 0 days (*n*=101)	Overall planned outpatients (*n*=1935)
- **Acute kidney injury**			
no	1831 (99.8%)	99 (98.0%)	1930 (99.7%)
yes	3 (0.2%)	2 (2.0%)	5 (0.3%)
- **Deep vein thrombosis**			
no	1834 (100%)	101 (100%)	1935 (100%)
yes	0 (0%)	0 (0%)	0 (0%)
- **Venous thromboembolism requiring therapy**			
no	1834 (100%)	101 (100%)	1935 (100%)
yes	0 (0%)	0 (0%)	0 (0%)
- **Superficial incisional surgical site infection**			
no	1827 (99.6%)	99 (98.0%)	1926 (99.5%)
yes	7 (0.4%)	2 (2.0%)	9 (0.5%)
- **Deep incisional surgical site infection**			
no	1834 (100%)	101 (100%)	1935 (100%)
yes	0 (0%)	0 (0%)	0 (0%)
- **Organ space surgical site infection**			
no	1834 (100%)	101 (100%)	1935 (100%)
yes	0 (0%)	0 (0%)	0 (0%)
- **Wound disruption**			
no	1834 (100%)	101 (100%)	1935 (100%)
yes	0 (0%)	0 (0%)	0 (0%)
- **Systemic sepsis**			
no	1831 (99.8%)	101 (100%)	1932 (99.8%)

	Outpatients = 0 days (*n*=1834)	Outpatients > 0 days (*n*=101)	Overall planned outpatients (*n*=1935)
yes	3 (0.2%)	0 (0%)	3 (0.2%)
**- Urinary tract infection**			
no	1823 (99.4%)	97 (96.0%)	1920 (99.2%)
yes	11 (0.6%)	4 (4.0%)	15 (0.8%)
**- Discharge to post-acute care**			
no	1750 (95.4%)	90 (89.1%)	1840 (95.1%)
yes	84 (4.6%)	11 (10.9%)	95 (4.9%)
**Telephone follow-up status day 30:**			
Alive	1745 (95.1%)	95 (94.1%)	1840 (95.1%)
Dead	4 (0.2%)	0 (0%)	4 (0.2%)
Follow-up not performed	85 (4.6%)	2 (2.0%)	87 (4.5%)
Missing	0 (0%)	4 (4.0%)	4 (0.2%)
**Telephone follow-up day 30: Any complications after discharge**			
yes	15 (0.8%)	1 (1.0%)	16 (0.8%)
- Cardiac	3 (0.2%)	0 (0%)	3 (0.2%)
- Pulmonary	7 (0.4%)	1 (1.0%)	8 (0.4%)
- Stroke	1 (0.1%)	0 (0%)	1 (0.1%)
- Acute kidney injury	5 (0.3%)	0 (0%)	5 (0.3%)
no	1776 (96.8%)	95 (94.1%)	1871 (96.7%)
Missing	43 (2.3%)	5 (5.0%)	48 (2.5%)

Data are presented as n (%).
