# Supplementary material for: Characteristics and outcomes of older patients undergoing out‐ versus inpatient surgery in Europe. A secondary analysis of the Peri‐interventional Outcome Study in the Elderly (POSE)
Source: Acta Anaesthesiol Scand. 2025 Mar 24;69(4):e70021. doi: 10.1111/aas.70021 (PMC11932067; doi:10.1111/aas.70021)
Supplement: Supplementary file 9 — Data S1: POSE‐study group. [file AAS-69-0-s008.pdf]

POSE-study group Collaborators

Alina Schenk
Ralf-Dieter Hilgers
Federico Bilotta
Leo C Bollheimer
Wolfgang Buhre
Ulf Guenther
Andreas Hoeft
Peter Lee
Idit Matot
Steffen Rex
Jacob Steinmetz
Jos Tournoy
Zekeriyya Alanoglu
Marc M Berger
Xavier Falières
Nicolai Goettel
Andrijan Kartalov
Konstantinos Katsanoulas
Jakub Kenig
Victoria Khoronenko
Lars H Lundstrøm
Tamar Macharadze
Miodrag Milenovic
Serge Molliex
Rosário Órfão
Marina Soro
Mihai Stefan
Zerrin Sungur
Tamas Szakmany
Victoria Baños
Mireia Rodriguez
Selene Martinez
Thomas Saller
Simon T Schäfer
Edouard Clermond
Charlotte Martin
Charlene Le Moal
Frederik Staikowsky
Bertand Delannoy
Olivier Desebbe
Carlo Missant
Matthias Desmet
Hans-Joerg Gillmann
Thomas Stueber
Sille M Dalsø
Morten Vester-Andersen
Andreas Ranft

Gerhard Schneider
Christel Huygens
Roselien Meeusen
Patricia Cruz
Carmen Fernández
Mareike Otto
Agathe Giltaire
Pascal Hofmann
Simone Gurlit
Alejandro Romero Fernández
Federica Castelli
Alexandre Ntouba
Julien Lanoiselée
Regina Schulz
Mathias Opperer
Julia Van Waesberghe
Sebastian Ziemann
Einat Refaeli-Awin
Zuleyha Kazak Bengisun
Immanuel Buchman
Dana Yahav-Shafir
Antonia Dimakopoulou
Morgan Le Guen
Aurelio Rodríguez-Pérez
Maud Beran
Aurelien Bonnal
Matthias Garot
Olivier Maupain
Denis Michel
Sofia Fernandes
Maria Sanabra
Eitan Mangoubi
Emmanuel Boselli
Timothy Switzer
Jose I García-Sánchez
Matthieu Boisson
Konstantinos Stamoulis
María Merino García
Hinnerk Wulf
David Gouraud
Christophe Lebrun
Sigismond Lasocki
Luzius A Steiner
Lars Bergmann
Bertram Baenziger
Georgios Karpetas
Basak C Meco
Ayşe Hızal
Rosa Méndez Hernández

Valerie Smit-Fun
Pedro Charco
Frank Nickel
Laura Grau Torradeflot
Marc Berger
Helmut Farcher
Mathias Opperer
Ine Adriaensens
Vera Saldien
Johan Berghmans
Sofie Van Hove
Maud Beran
Gert-Jan Eerdekens
Dieter Mesotten
Maxim Timmers
Elly Vandermeulen
Ann De Bruyne
Stefan De Hert
Hendrik De Ruyter
Vincent Van Belleghem
Isabelle Boscart
Wouter De Corte
Matthias Desmet
Carlo Missant
Stefaan Carlier
Charlotte Castelain
Caroline Demeyer
Carl Vandenbossche
Carlo Missant
Hans Detienne
Sarah Devroe
Geertrui Dewinter
Danny Hoogma
Christel Huygens
Roselien Meeusen
Steffen Rex
Marc Van de Velde
Christophe Lebrun
Stéphanie Poels
Filiep Soetens
Christian Fenger-Eriksen
Christina Draegert
Sofia Gaspar Santos
Christine Soelling
Jacob Steinmetz
Gertrud Andersen
Sille M Dalsø
Pernille Haderslev
Vibe M Rasmussen

Morten Vester-Andersen
Tine G Sommer
Johan Kirkegaard
Lars H Lundstrøm
Christian M Olesen
Sansu Paramanathan
Lisbet Tokkesdal Jensen
Halfdan H Knudsen
Jens C Schmidt
Nick P Stehen
Hervé Dupont
Clément Herbinet
Emmanuel Lorne
Yazine Mahjoub
Alexandre Ntouba
Marine Fritsch
Manuela Garcia
Sigismond Lasocki
Jonathan Petit Phan
Thomas Lieutaud
Laura Bonneric
Emmanuel Boselli
Maxime Gaillet
Marc Danguy des Déserts
Etienne Montelescaut
Antoine Lamblin
Violaine Muller
Celine Lagrange
Charlene Le Moal
Alain Robert
Frederik Staikowsky
Benoit Lebas
Gilles Lebuffe
Matthias Garot
Johanne Beuvelot
David Dejour
Emmanuel Deligne
Olivier Desebbe
Bertand Delannoy
Benoit Gignoux
Olivier Guillaud
Joseph Nloga
Florence Prunier-Bossion
Franck Sibellas
Paul Abraham
Cyril Bidon
Thomas Rimmele
Marie-Hélène Bruge-Ansel
Arnaud Friggeri

Anne-Claire Lukaszewicz
Mikhail Dziadzko
Marc Leone
Zoe Meresse
Bruno Pastene
Isabelle Odin
Aurelien Bonnal
Nicolas Bouic
Pierre Trinh Duc
Thomas Pillant
Fabien Riboulet
Samuel Degoul
Nicolas Saumier
Marion Wasilewski
Karim Asehnoune
Antoine Roquilly
Pauline Glasman
Louis Puybasset
Fanny Garnier
Franck Verdonk
Charles M Samama
Line Towa
Alice Blet
Stéphanie Barrau
Matthieu Boisson
Bertrand Debaene
Denis Frasca
Nadia Imzi
Bernard Delvaux
Davy Huynh
Olivier Maupain
Luc Mercadal
Nabil Zanoun
Armelle de Baene
Catherine Boulay-Maninovsky
Olivier Fernandes
Agathe Giltaire
Philippe Gomis
Jean-Marc Malinovsky
François-Xavier Romain
Astrid Calmelet
Ségolène Dupont
David Gouraud
Sophie Millet
Frédéric Simonneau
Francoise Charret
Charlène Couturier
Julien Lanoiselée
Estelle Lornage

Jeremy Mallard
Ryan Milati
Sylvie Passot
Sylvain Vallier
Mihaela L Agavriloaia
Quentin Badoux
Mehdi Lewandowski
Yanis Mermet
Denis Michel
Olga Kiskira
Sherifa Adjavon
Virginie Dumans
Morgan le Guen
Julien Josserand
Sabrina Ma
Jeremy Castanera
Benjamin Massiera
Philippe Petua
Fanny Bounes-Vardon
Gaëlle Bosc
Laëtitia Bosch
Edouard Clermond
Fabrice Ferre
François Labaste
Charlotte Martin
Rémi Menut
Vincent Minville
Mohamed Srairi
Maria Tarasi
Florent Varin
Société Française d'Anaesthésie et de Réanimation (SFAR) Research Network, Paris
Mark Coburn
Ana Kowark
Linda Grüßer
Rolf Rossaint
Julia Van Waesberghe
Sebastian Ziemann
Lars Bergmann
Hartmuth Nowak
Günther Oprea
Katharina Rump
Matthias Unterberg
Heike Vogelsang
Mitja Klutzny
Claudia Neumann
Martin Soehle
Maria Wittmann
Martin Scharffenberg

Jakob Wittenstein
Jonas Hinterberg
Peter Kienbaum
Giovanna Lurati-Buse
Frank Nickel
Maximilian Schäfer
Simone Lindau
Patrick Meybohm
Hans-Joerg Gillmann
Florian Piekarski
Theresa A Kaufhold
Wolfgang Koppert
Andreas Leffler
Hans-Peter Reiffen
Diana Rudolph
Henning Starke
Thomas Stueber
Petra Bischoff
Heinz Haberecht
Heiko Plehn
Michael Bauer
Andreas Kortgen
Christoph Sponholz
Uwe Krüger
Sabine Müller-Esch
Mareike Otto
Christian Rempf
Christian Schmidt
Dunja Schumacher
Juliane Blazek
Christin Büttner
Andrea Leibeling
Dirk Rüsch
Hinnerk Wulf
Karsten Burow
Eugen A El-Hilali
Christian Greke
Paul Großmann
Mario Kluth
Regina Schulz
Sofiane Dridi
Ivana Popovska
Andrés Brenes
Andreas Ranft
Pia Feddersen
Dominik Gerstmeyer
Philippe Fthenakis
Gerhard Schneider
Dirk Miketta

Vera von Dossow
Philipp Groene
Dominik Höchter
Klaus Hofmann-Kiefer
Tobias Kammerer
Malte Kamrath
Thomas Saller
Simon T Schaefer
Roland Tomasi
Tobias Wiedemann
Catharina Zeuzem-Lampert
Bernhard Zwissler
Stephan Braune
Mona Brune
Simone Gurlit
André Hemping-Bovenkerk
Michael Möllmann
Mario Santamaria
Leonie M Schirwitz
Melanie Meersch
Alexander Zarbock
Ulf Guenther
Stefanie Decker
Berthold Drexler
Silvia Hipp
Pascal Hofmann
Markus Müller
Judith Roth
Miriam Seiß
Christian Adam
Ingo Schwartges
Peter Kranke
Konstantinos Katsanoulas
Pelagia Chloropoulou
Antonia Andreeva
Antonia Dimakopoulou
Amalia Douma
Iphigeneia Gregoriadou
Evelina Koutsouli
Konstantina Mendrinou
Eirini Mavrommati
Anastasios Stathopoulos
Chrysanthi Batistaki
Paraskevi Matsota
Konstantina Kalopita
Vasiliki Skandalou
Marina Balanika
Georgios Papathanakos
Petros Tzimas

Evgenia Ketikidou
Anastasia Vachlioti
Bioulent Kiamiloglou
Evangelia Nikouli
Eleni Arnaoutoglou
Konstantina Kolonia
Eleni Laou
Konstantinos Stamoulis
Epaminondas Vlachakis
Georgios Karpetas
Ioanna Lianou
Maria Spyraki
Irini Tatani
Eleni Panagiotou
Evangelia Samara
Anna Kolesnikova
Freideriki Sifaki
Eirini Zarzava
Athanasios Bampzelis
Eleni Georgopoulou
Eleni Christidou
Georgia Tsaousi
Maria Nastou
Orestis Ioannidis
Eugene Dolzenko
Georgia Geleve
Eleni Logotheti
Fotios Yfantidis
Peter Lee
Senbagam Rajamanickam
Shanmuga Ramaswamy
Timothy Switzer
Gurmukh Das Punshi
Karthikeyan Srinivasan
Michael Gilmartin
Osmond Morris
Immanuel Buchman
Yaacov Gozal
Amar Merissat
Reut Peled
Dafna Willner
Hila A Chariski
Leonid A Eidelman
Michal Y Livne
Eitan Mangoubi
Haim Berkenstadt
Dina Orlcin
Dana Yahav-Shafir
Rita Aharonov

Anat Cattan
Lior Felman
Idit Matot
Einat Refaeli-Awin
Yohai Steinberg
Wisam Zabeeda
Andrijan Kartalov
Biljana Kuzmanovska
Filip Naumovski
Marija Toleska
Atanas Sivevski
Xavier Falières
Anouk Andriessen
Minke Kortekaas
Wolfgang Buhre
Roos Van Gorp
Dianne de Korte-de Boer
Valerie Smit-Fun
Maurice Theunissen
Mirjam Droger
Toine van den Enden
Seppe Koopman
Marije Marsman
Eva van Schaik
Jakub Kenig
Marta Azenha
Camile Lanzaro
Rosário Órfão
Andreia Borrego
Pedro Branquinho
Sofia Fernandes
Miguel Laires
Denise de Noronha
Inês Ferraz
Ana Pires
Joana Silva
Dan Corneci
Oana Oprea
Stefan-Vladimir Zahiu
Dana R Tomescu
Ioana M Grintescu
Daniela Filipescu
Mihai Stefan
Elena Stefanescu
Andrey Vazenin
Danil Baskakov
Victoria Khoronenko
Dmitry Tipisev
Ksenia Kozlova

Olivera Marinkovic
Ana Sekulic
Miodrag Milenovic
Marija Rajkovic
Marija Djukanovic
Jovanka Nikolic
Svetlana Sreckovic
Marina Stojanovic
Nebojsa Ladjevic
Jelena Jovicic
Dragana Unic-Stojanovic
Biljana Stosic
Aleksandra Bulasevic
Marina Soro
Alma M Espinosa-Moreno
Jose I García-Sánchez
Beatriz Martín-Vaquerizo
Clara Morandeira-Rivas
Diana Zamudio
Victoria Baños
Mireia Rodriguez
Selene Martinez
Nerea Guadalupe
Gracia Herranz
Javier Baute
Vanesa Madrona
Roser de Jose
Jordi Miralles
Alfred Merten
Rolando Muñoz
Anabel Delgado
Victoria Moral
Aleix Carmona Blesa
Sara Espejo
Laura Grau Torredeflot
Alejandro Romero Fernández
Maria Sanabra
Pere Serra Pujol
Maria J Alvira Uribe
Astrid Alvarez Perez
Espedito Brunetto
Federica Castelli
Jorge Gonzalez Aguirre
Adriana Herivas Villar
Guido Munoz Rojas
Aleix Carmona Blesa
Natalia Montero
Víctor Baladrón González
Ángel Becerra-Bolaños

Aurelio Rodríguez-Pérez
Luis Santana-Ortega
Vanessa Suárez-Romero
María L Torres-Machí
Javier Ferrero de Paz
Jose M Marcos -Vidal
Ana Martín Garcia
María Merino García
Consuelo Rego Diaz
Ana Crespo Santiago
Lourdes Ferreira Laso
Felix Lobato Solores
Alba Burgos
Alberto Calvo
Patricia Cruz
Carmen Fernández
Ignacio Fernández
Ignacio Garutti
Fernando Higuero
David Martinez
Patricia Piñeiro
Sonia Expósito Carazo
Rosa Méndez Hernández
Mar Orts Rodríguez
Fernando Ramasco Rueda
Ane Abad-Motos
Javier Ripollés-Melchor
Carmen Pastor López
Pedro Charco
Sara Perez-Palao
Laura Sancho-Iñigo
Nasara Segura
Marina Soro
Esther Utrera
Ania Albinarrate
Ana M Fondarella
Lucia Gallego-Ligorit
Luisa Lacosta Torrijos
Oliver Bandschapp
Andrea A Blum
Nicolai Goettel
Esther Seeberger
Luzius A Steiner
Alessandra E Thomann
Seraina Frei
Susan Hoehn
Bertram Baenziger
Giuliana Capaldo
Daniel Christ

Ramon Doerig
Daniel Hodel
Andreas Weiss
Lukas Witt
Philippe Schumacher
Dirk A Siebing
Zerrin Sungur
Zekeriyya Alanoglu
Seyma Orcan Akbuz
Zuleyha Kazak Bengisun
Baturay K Kazbek
Ulku C Koksoy
Engin Z Terzi
Hakan Yilmaz
Neslihan Alkis
Sanem Cakar Turhan
Basak C Meco
Konul Hajiyeva
Cigdem Yildirim Guclu
Jülide Ergil
Emine Unal Ceran
Menekse Ozcelik
Atik Bülent
Kilinc Gökhan
Kemal T Saracoglu
Bunyamin Kir
Kemalettin Koltka
Nükhet Sivrikoz
Pelin Corman Dincer
Nur Canbolat
Turkan Kudsioglu
Gaye Aydin
Ceren Aygün Mucuoglu
Duriye G Inal
Semih Kucukguclu
Ayse I Egilmez
Betul Kozanhan
Munise Yildiz
Hüseyin U Pinar
Başar Erdivanlı
Ayşe Hizal
Emre Karagöz
Hızır Kazdal
Abdullah Özdemir
Ayca Tas Tuna
Gamze Gulgun
Dolya Oleg
